# Effects of NK cell-related lncRNA on the immune microenvironment and molecular subtyping for pancreatic ductal adenocarcinoma

**DOI:** 10.3389/fimmu.2024.1514259

**Published:** 2025-01-13

**Authors:** Jinze Li, Chuqi Xia, Yuxuan Li, Hanhan Liu, Cheng Gong, Daoming Liang

**Affiliations:** ^1^ Department of Gastrointestinal Surgery, The Second Affiliated Hospital of Kunming Medical University, Kunming, Yunnan, China; ^2^ Department of Pathology, Maternal and Child Health Hospital of Hubei Province, Tongji Medical College, Huazhong University of Science and Technology, Wuhan, China; ^3^ Department of Hepatobiliary and Pancreatic Surgery, Zhongnan Hospital of Wuhan University, Wuhan, China

**Keywords:** tumor immune single-cell hub 2, pancreatic ductal adenocarcinoma, NK cell-related lncRNAs, tumor immune microenvironment, molecular subtyping

## Abstract

**Background:**

Patients with pancreatic ductal adenocarcinoma (PDAC) face a highly unfavorable outcome and have a poor response to standard treatments. Immunotherapy, especially therapy based on natural killer (NK) cells, presents a promising avenue for the treatment of PDAC.

**Aims:**

This research endeavor seeks to formulate a predictive tool specifically designed for PDAC based on NK cell-related long non-coding RNA (lncRNA), revealing new molecular subtypes of PDAC to promote personalized and precision treatment.

**Methods:**

Utilizing the Tumor Immune Single-cell Hub 2 platform, we discovered genes associated with NK cells in PDAC. We employed the TCGA-PAAD dataset to ascertain the expression profiles of these NK cell-related genes and to screen for lncRNAs correlated with NK cells. Subsequently, we utilized Cox regression analysis for hazard ratios and LASSO regression analysis to identify three NK cell-related lncRNAs that were used to develop a prognostic assessment model. The forecasting accuracy of this model was appraised using the ROC curve and validated using a test set and the complete dataset.

**Results:**

Successful construction of a prognostic model comprising three lncRNAs was achieved, demonstrating good predictive efficiency in the training set, validation dataset, and the entire dataset. NK cells display robust interactions with malignant cells, CD8 T cells, and fibroblasts in the PDAC tumor microenvironment and participate in the transport of various signaling molecules and following immune responses in PDAC. According to the expression patterns of NK cell-related lncRNA, we labeled PDAC patients as four molecular subtypes, exhibiting significant differences in immune cell infiltration, drug sensitivity, and other aspects.

**Conclusion:**

This study Uncovered the activity of NK cells within PDAC, proposed an NK cell-related lncRNA model, and delineated new molecular subtypes, thereby providing targets for personalized therapy.

## 1 Introduction

Pancreatic cancer is the sixth most frequent cause of death attributed to cancer worldwide, responsible for approximately 5% of all cancer deaths. In 2022, approximately 511,000 individuals were diagnosed with new pancreatic cancer cases, and 467,000 succumbed to the disease, characterized by the most unfavorable prognosis among all tumors (1). Pancreatic ductal adenocarcinoma (PDAC) represents the majority of pancreatic cancer diagnoses, exceeding 90% (2). It exhibits an exceptionally high degree of malignancy, as 80% to 85% of patients are already in an advanced, unresectable period at the date of clear diagnosis. Moreover, PDAC generally exhibits resistance to most chemotherapy drugs (3). Resistance to conventional therapeutic approaches has resulted in a persistent lack of improvement in survival rates over the past few decades. The advancing field of immuno-oncology may offer a breakthrough for enhancing the prognosis and potentially curing PDAC. Immunotherapies primarily comprise checkpoint inhibitors and adoptive cell therapies, which function primarily through modulating the immune reaction to perceive and battle cancer cells. Studies have indicated that the combination of chemotherapy and PD-1 antibodies has improved the overall survival rate among PDAC individuals (4).

Although the use of immunotherapy in PDAC patients faces a few challenges, including the “cold” tumor microenvironment (TME), which is marked by myeloid cell aggregation, a dearth of CD8+ T cells, and minimal activation marker expression, these factors suggest a significant impairment or absence of adaptive T cell immunity (5). Research indicates that targeted strategies, including the enhancement of co-stimulatory signals, the application of checkpoint inhibitors, and the use of cytokines to augment the activity and longevity of NK cells, may substantially improve survival rates among PDAC patients (6). An intense focus on T cells has resulted in the undervaluation of other immune cells’ potential within the TME. Despite their integral role in the body’s defenses, NK cells have not been as thoroughly investigated in tumor immunotherapy. Nonetheless, NK cells possess distinctive benefits in combating tumors, thereby cementing their importance in this domain. Their capacity for swift response and immediate attack initiation, lack of major histocompatibility complex (MHC) restrictions, broad target recognition, reduced autoimmunity risk, and their availability for genetic engineering highlight their therapeutic potential. The efficacy of NK cell immunotherapy, both as a monotherapy and in conjunction with other treatment modalities, has been demonstrated (7). Thus, further investigation into the distinctive function of NK cells in PDAC and their synergistic interactions with other immune cells is essential, holding substantial promise for the reprogramming of the tumor immune microenvironment and for advancing PDAC treatment strategies.

Long non-coding RNAs (lncRNAs), surpassing 200 nucleotides, belong to the non-coding RNA family, and function as pivotal regulatory elements. They exhibit diverse roles in the critical biological processes of PDAC (8). Research has demonstrated that prognosis models predicated on lncRNAs associated with immune responses have accurately forecasted survival outcomes in patients with ovarian cancer (9). lncRNA biomarkers that are associated with immune functions are invaluable for assessing the survival rates of patients with hepatocellular carcinoma (HCC) (10). Immune-infiltration-associated lncRNA models hold prognostic significance and can predict therapeutic responses among individuals affected by non-small cell lung cancer (NSCLC) (11). However, the study of NK cell-associated lncRNA in PDAC immunotherapy has not been reported. The NK cell-associated lncRNAs may offer a novel target for developing immunotherapeutic strategies against PDAC.

In our investigation, gene enrichment analysis revealed the multifaceted immunomodulatory functions of NK cells in PDAC. Utilizing the Tumor Immune Single-Cell Hub 2 (TISCH2) database, we pinpointed distinguishingly expressed NK cell-related genes (NKGs) in PDAC. We then leveraged The Cancer Genome Atlas Pancreatic Adenocarcinoma Collection (TCGA-PAAD) to ascertain the expression profiles of these NKGs, and through correlation analysis, identified lncRNAs correlated with NK cells. Subsequently, we employed a univariate Cox regression model, which was then complemented with the least absolute shrinkage and selection operator(LASSO) regression to refine the selection of variables. These were complemented with multivariate Cox regression analysis to meticulously construct a prognostic model incorporating three NK-related lncRNAs. The efficacy of this model was substantiated by applying both a validation cohort and the complete set. Further independent prognostic analyses verified that the risk score derived from our NK-related lncRNA prognostic model serves as a separate indicator of prognosis for PDAC.

Our independent prognostic analysis, complemented by ROC curves, demonstrated that the risk score, as a solitary variable for prognosis evaluation, markedly surpassed other clinical indicators in predictive accuracy. Through functional enrichment analysis, we unveiled the potential regulatory mechanisms of NK cell-related lncRNAs in PDAC. Analysis of tumor mutational burden illuminated the variance in mutated gene frequency among disparate risk groups. Furthermore, our immune-related analyses elucidated the likely immune regulatory roles of NK cell-related lncRNAs in PDAC. Not only that, we established four novel molecular subtypes of PDAC based on the NK cell-related lncRNA prognostic model. This stratification is instrumental in advancing precise and personalized clinical therapies, offering tangible guidance for clinical decision-making.

## 2 Materials and methods

### 2.1 Data collection and preprocessing

The scRNA details for PDAC were acquired from the GSE162708 dataset of the Gene Expression Omnibus website (GEO), consisting of 22,133 cells. For the analysis of this single-cell data, we employed the TISCH2 platform (12). We utilized Principal Component Analysis (PCA) to diminish the data’s dimensionality, the formula is: 1. euclidean Distance: $\left(x,X_{i}\right)=\sqrt{\sum_{j=1}^{d}{\left(x_{j}-X_{ij}\right)}^{2}}$ . 2. the Manhattan Distance: $d\left(x,y\right)=\sum_{i=1}^{n}\left|x_{i}-y_{i}\right|$ .Followed by the application of K-nearest neighbors and Louvain algorithms for the recognition and classification of distinct cell populations, the formula is: 1. Modularity Gain: $\text{Δ}Q=\frac{1}{2m}\sum_{i\in C}\left(\frac{k_{i,in}^{2}}{m}-\gamma \frac{k_{i}^{2}}{m^{2}}\right)$ . 2. modularity gain of directed graph: $\text{Δ}Q=\frac{1}{m}\left(k_{i,in}-\gamma k_{i,out}\right)\cdot \left(\sum_{tot,in}+k_{i,in}\cdot \sum_{tot,out}\right)$ . To annotate cell types, we relied on specific marker genes. The Wilcoxon test was subsequently employed to identify genes that were significantly differentially expressed within the NK cell population relative to all other cell populations. We set a log fold change threshold of at least 1.5 and a target FDR of less than 0.05 as the criteria for screening (13).

### 2.2 Cell-to-cell communication analysis

We employed the version1.0.0 Cell Chat tool on the TISCH2 (14) platform to scrutinize the gene patterns of identified ligand-receptor (L-R) pairs across various cell populations, thereby evaluating cellular interactions. The tool is based on a mass interaction model via the Hill function: $f\left(x\right)=\frac{x^{n}}{K^{n}+x^{n}}$ . The default critical value for the interaction score is 0.01. To quantify and visualize the number of substantial L-R interactions and the connection probabilities between distinct cellular communities. By using the netVisual_circle tool provided by the R.pheatmap package in tandem with R.CellChat, we proceeded with the visualization. Each community was analyzed for significant L-R pairs, which were classified as donors or recipients, with a significance threshold of P-value less than 0.05 (12).

### 2.3 Functional enrichment analysis of multiple cell populations

To explore the enrichment characteristics of each group of cells, we performed gene set enrichment analysis (GSEA) utilizing the TISCH2 platform (12), we sorted the genes according to their fold change from the differential analysis. Utilizing Kyoto Encyclopedia of Genes and Genomes (KEGG) analysis, Gene Ontology (GO) enrichment analysis, and GSEA, significantly altered biological pathways were identified and demonstrated across cell populations (designating 0.05 as the cutoff for FDR), which helped us to acquire a further insight into the functional enrichment of different cell populations (12).

### 2.4 Collection of PDAC transcriptome and clinical data

We assembled gene expression information, clinical profiles, and data on somatic mutations from 179 tumor samples and 4 normal pancreatic tissues within the TCGA database. Following this, we carried out a differential expression analysis comparing mRNA expression levels in tumor versus non-tumor tissues. By integrating the NK cell-related differential genes previously filtered and applying a correlation coefficient threshold of 0.4, we identified 3,491 lncRNAs correlated with NK cells. For the gene expression data analysis, we employed the “R.limma” package (15), We identified 304 lncRNAs associated with NK cells that exhibited significant differential expression between the tumor and non-tumor groups.

### 2.5 Model construction and validation

Within this research, patients with PDAC were evenly divided into two groups at a ratio of 1:1, designated as the training dataset and the validation set. We utilized a single-variable Cox model to pinpoint 42 NK cell-related lncRNAs associated with PDAC prognosis within the training set. The COX regression formula is: $h\left(t\right)=h_{0}\left(t\right)\exp\left(\beta_{1}x_{1}+\beta_{2}x_{2}+\ldots +\beta_{k}x_{k}\right).$ To mitigate the risk of model overfitting, LASSO regression analysis was applied. The formula is: $\underset{\beta }{\min}\left\{\frac{1}{2n}\left|y-X\beta {|}_{2}^{2}+\lambda \right|\beta {|}_{1}\right\}$ . Subsequently, a predictive model for PDAC prognosis was constructed utilizing multivariate Cox regression on the training set. The model-assigned coefficients for each NK cell-related lncRNA allowed us to derive the risk score formula: risk score = 0.456120265000581 * LINC00519 expression - 1.12039497572332 * PAN3-AS1 expression + 0.479223014745653 * LINC02004. Based on risk scores, patients were segregated into two groups: high-risk and low-risk. The Kaplan-Meier method was then applied to compare survival between these risk groups. The efficacy of the model was tested using the ROC curve method, and the test set’s performance was further validated against the validation set and the entire dataset (16).

### 2.6 Comparative expression and functional profiling analyses across risk groups

With the help of the R. limma package, we discern genes with differential expression (DEGs) across various risk groups, applying stringent selection criteria of (|logFC| > 1, FDR < 0.05). The differential gene analysis outcomes were graphically represented through volcano plots and heat maps for clear visualization. To probe more deeply into the functional aspects of these DEGs, we exploited the clusterProfiler R package to carry out Gene Ontology (GO) analysis, which encompassed the three domains: biological process (BP), cellular component (CC), and molecular function (MF). Furthermore, we analyzed the KEGG pathway, which pathways that were significantly enriched were graphically depicted using bubble charts for intuitive understanding. Furthermore, GSEA was applied to evaluate the disparities in biological functions between different risk groups, with the thresholds set at (|normalized enrichment score| > 1, FDR < 0.05).

### 2.7 Tumor mutational burden and tumor immune infiltration correlation analysis

Within the scope of this study, we harnessed the maftools package in R for the visualization and analytical assessment of tumor mutational burden (TMB). This robust tool facilitated the visualization and quantification of mutation frequency and quantities within tumor samples. Furthermore, we utilized a suite of computational tools, including EPIC, TIMER, XCELL, MCPCOUNTER, QUANTISEQ, CIBERSORT-ABS, and CIBERSORT, to quantify the levels of immune cells present in the tumor tissue in the TCGA-PAAD samples. These analyses provided insights into the immunological microenvironment and its potential implications for therapeutic strategies (17). Concurrently, an immune checkpoint analysis between different risk groups was performed by R. limma and R. reshape 2. Finally, we conducted immune-related functional analysis employing the ssGSEA method through the R.GSVA package, which allowed us to assess the enrichment of immune-related pathways and functions across various risk groups.

### 2.8 Tumor subtyping based on the model

We employed consensus clustering analysis within an unsupervised learning framework, utilizing the ConsensusClusterPlus package (18), to delineate molecular subtypes of PDAC. The package generated critical visualizations: the consensus cumulative distribution function (CDF) plot, the consensus matrix (CM), and the consensus heatmap. These tools were instrumental in establishing the ideal cluster count for PDAC subtypes, while the CDF plot ensured the stability of diverse clustering setups, the CM detailed the frequency of sample clustering in a matrix format across multiple iterations, and the consensus heatmap offered a visual synopsis of the CM, simplifying the interpretation of clustering outcomes. Collectively, these analyses facilitated the identification of the most appropriate number of classifications for PDAC subtypes, as per the prognostic risk model.

### 2.9 Cell culture

Human pancreatic cancer cell lines BXPC-3, PANC-1, SW1990, ASPC1, COLO357, and human pancreatic normal cell line HPDE6-C7 were purchased from Shanghai Yaji Bio. All cell lines were cultured in RPMI-1640 medium (Sigma-Aldrich, St. Louis, MO, USA) supplemented with 10% fetal bovine serum (Gibco, USA) and 1% penicillin-streptomycin-glutamine (PSG; Thermo Fisher Scientific, Dreieich, Germany), at a temperature of 37°C with 5% CO2 to eliminate the interference of culture conditions.

### 2.10 Real-time quantitative PCR

Total RNA was extracted using the Trizol method (T9108, Takara, Dalian, China), and reverse transcription was performed using an enzyme kit. Subsequently, qRT-PCR was conducted using 2 × ChamQ Universal SYBR qPCR Master Mix (Q711-02, Vazyme, Nanjing, China). The primer sequences used are as follows: LINC00519 Forward: ATGGAAAGTGAGGGCAGACAC; Reverse: GCCCTTTGAAGCATTTCTCCAG. LINC02004 Forward: AGAGCAGCACAGTGAGTCAG; Reverse: CAGTGCTGGGCTATCCTGAA. PAN3-AS1 Forward: AAATTCTGCCTCCACTCGCTC; Reverse: CTACCCATAAGCCCTCGCGT. GAPDH Forward: GGAGCGAGATCCCTCCAAAAT; Reverse: GGCTGTTGTCATACTTCTCATGG.

### 2.11 Transfection

The overexpression plasmids pcDNA3.1-LINC00519, pcDNA3.1-LINC02004, and pcDNA3.1-PAN3-AS1 were obtained from Wuhan Qing Ke Biological Company. Cells were seeded into 6-well plates at the correct density. After 24 hours at 37°C, transfection was performed using Lipofectamine 3000 (Sigma, USA) according to the manufacturer’s instructions, and the cells were then used for subsequent experiments.

### 2.12 Colony formation experiment

After transfection for 24 hours, 500 cancer cells were evenly distributed into each well of a 6-well culture plate and cultured under conditions of 37°C and 5% CO2 until visible colonies formed. At the end of the culture period, the cells were fixed with a 10% formaldehyde solution for 5 minutes, followed by staining with 0.1% crystal violet for 1 hour. After two weeks of cultivation, the formed colonies were counted. To enhance the reliability of the data, the experiment was repeated three times, and the results were averaged.

### 2.13 Transwell invasion assay

Cells were seeded onto the upper chamber of a Transwell insert pre-coated with Matrigel, using serum-free medium. The lower chamber was filled with medium containing a chemoattractant. After incubation, non-invading cells on the upper surface of the membrane were removed, and invading cells on the lower surface were fixed and stained. Finally, the number of invaded cells was quantified using microscopy.

### 2.14 Statistical analysis

We carried out all statistical evaluations with R version 4.4.1. By using the Kruskal-Wallis test, we aimed to evaluate differences in immune checkpoint gene expression, immune scores, and drug sensitivity across various risk groups. The log-rank test, obtained from the R survival package, was utilized for conducting Kaplan-Meier survival analyses. The formula is: $\chi^{2}=\sum_{j=1}^{n}\frac{{\left(O_{j}-E_{j}\right)}^{2}}{E_{j}}$ . Additionally, the Cox model was applied in order to assess the collective impact of multiple variables. For all statistical analyses, a two-tailed test was used, with significance accepted at P-values less than 0.05. Significance levels were described as asterisks: *** denotes p < 0.001, ** denotes p < 0.01, and * denotes p < 0.05.

## 3 Results

### 3.1 NK cell-related immune microenvironment crosstalk in PDAC

**Figure 1:**
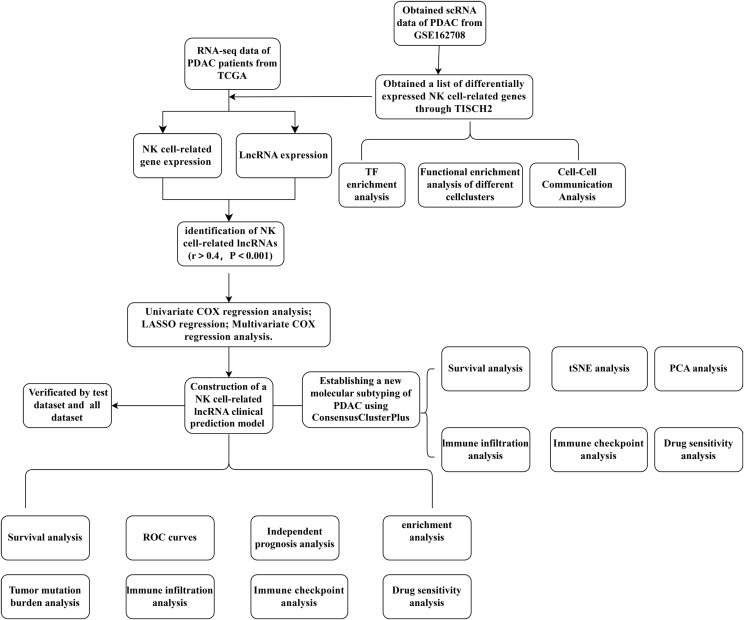
A detailed flowchart illustrating the construction, validation, and molecular subtyping of the NK cell-related lncRNA model in PDAC. PDAC, Pancreatic Ductal Adenocarcinoma; TISCH2, Tumor Immune Single-Cell Hub 2; TCGA, The Cancer Genome Atlas; TF, Transcription factor; LASSO, Least absolute shrinkage and selection operator; PCA, Principal component analysis; tSNE, T-distributed stochastic neighbor embedding; NK, Natural Killer cells.

**Figure 2:**
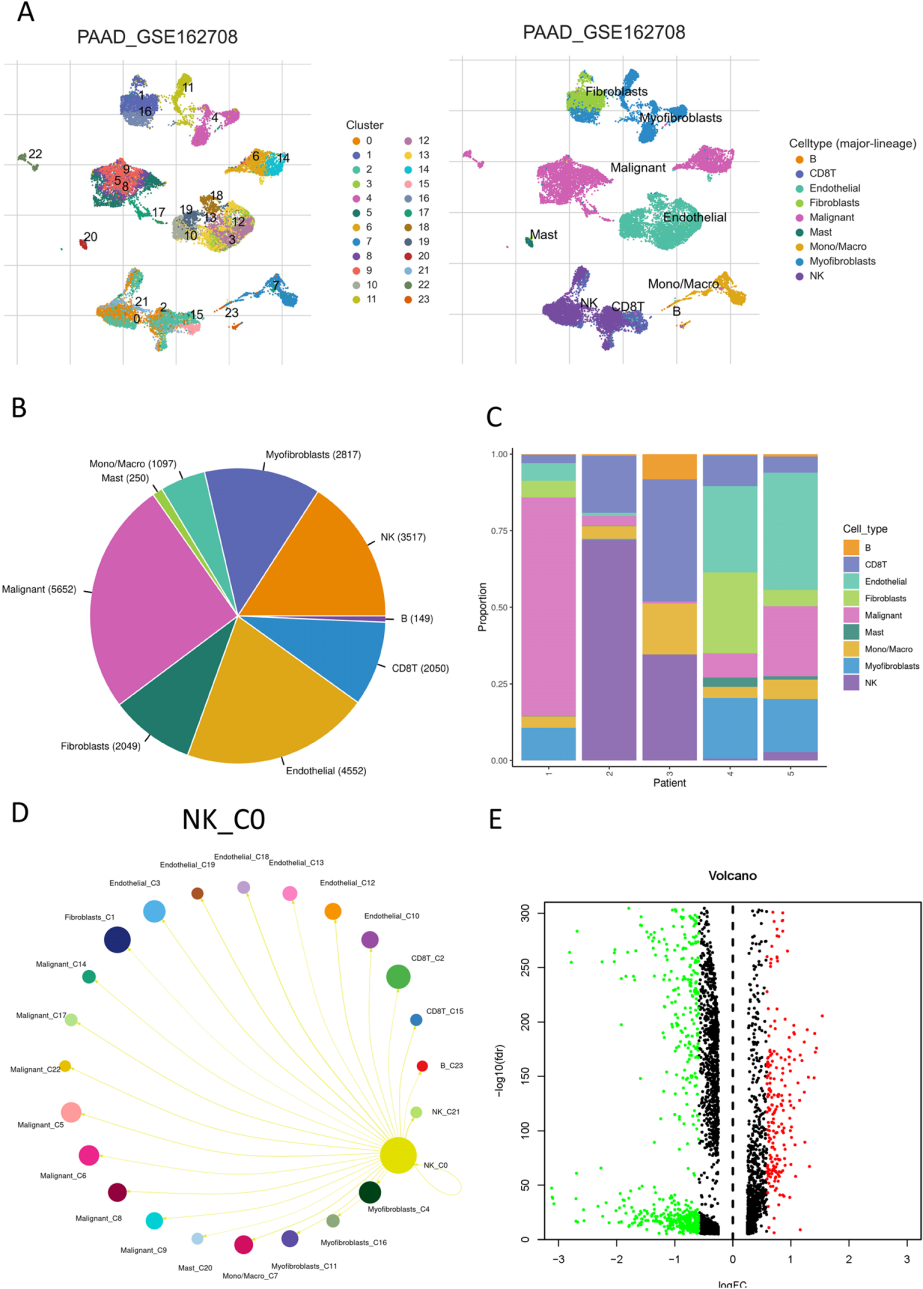
NK cell communication network in PDAC. **(A)** A UMAP plot displaying the distribution and abundance of different cell subsets in PDAC; **(B)** A pie chart showing the percentage of NK cells; **(C)** A bar graph illustrating the distribution of NK cells across individual samples; **(D)** The interaction probabilities between NK cells and other cells visualized through Cell Chat; **(E)** A volcano plot depicting differentially expressed genes within NK cells. Red indicates a fold change > 1.5, FDR < 0.05; green indicates a fold change < 1.5, FDR < 0.05.

**Figure 1** depicts a flowchart of the analysis and derivation pathway followed in this study. By visualizing the scRNA data of GSE162708 on TISCH2, **Figure 2A** distinctly illustrates the delineation of NK cell subsets. Through pie charts and bar graphs, we displayed the counts and relative frequencies of NK cells. The estimated total number of normal human NK cells is 2 × 10^10, accounting for approximately 1% of all immune cells in the body (19). Single-cell dataset analysis has found that in PDAC patients, NK cells constitute 15.9% of immune cells, significantly higher than the content of NK cells in normal human bodies, indicating an abnormal enrichment of NK cells in PDAC, which is of significant research importance (**Figures 2B, C**). Within the tumor immune microenvironment, cell-cell correlations are crucial for regulating cellular functions, immune status, and cancer development. Utilizing the Cell Chat method on the TISCH2 platform, we forecasted the interactions among diverse cell populations. The findings revealed robust interactions between NK cells and malignant cells, CD8 T cells, and fibroblasts (**Figure 2D**), with NK cells assuming a pivotal part in the PDAC immune microenvironment, modulating the tumor’s immune context through cellular crosstalk. We examined the genetic interactions between NK cells, either as signal transmitters (**Supplementary Figure S1A**) or signal receivers (**Supplementary Figure S1B**), and other cell types. The results revealed extensive genetic interactions between NK cells and varied other cell types, containing endothelial cells, malignant cells, CD8 T cells, and fibroblasts. To contrast NK cell-related genes with those of else cells, we applied the Wilcoxon test accessible in TISCH2. We pinpointed NK cell-related genes based on a log-transformed fold change (|fold change| > 1.5) and an FDR threshold below 0.05. The analysis yielded 387 upregulated and 699 downregulated NK cell-related genes (**Figure 2E**).

### 3.2 Functional characteristics of NK cells in PDAC

To clarify the possible regulatory functions of NK cells in PDAC and their interactions with other cellular components within the tumor immune microenvironment within the tumor immune microenvironment, we carried out GSEA using the TISCH2 platform. We observed robust correlations between NK cells and gene sets associated with CD8 T cells as well as B cells (**Supplementary Figure S2**). In the KEGG pathway enrichment analysis, NK cells were found to exhibit analogous regulatory patterns to B cells and CD8 T cells in biological processes such as natural killer cell-mediated cytotoxicity, primary immunodeficiency, ribosomal signaling, antigen preparation and presentation processes, as well as T cell receptor-mediated signaling (**Supplementary Figures S3**, **S4**). The GO analysis detected important enrichment of NK cells in several key biological processes, including cytoplasmic translational activities, activation of innate immune responses, and NK cell-mediated cytotoxicity. Moreover, NK cells demonstrated enrichment in specific cellular components like free ribosomes and RNA polymerase II, and in various molecular functions, including the formation of ribosomal structures, involvement in antigen and hormone binding, and modulation of the activity of membrane receptor tyrosine kinases (**Supplementary Figures S5**–**S7B**). In PDAC, a multitude of transcription factors assume a crucial role. Utilizing the spatial association algorithm on the TISCH2 platform, we deduced the pivotal transcription factors that orchestrate gene expression across various cell clusters. A heatmap graphically represents the high or low expression levels of core transcription factors within different cell clusters in the dataset. The transcription factor expression profile in NK cells closely mirrors that in monocytes/macrophages and CD8 T cells (**Supplementary Figure S7C**), hinting at a potential regulatory axis among these cell types that could influence PDAC progression. We then identified transcription factors that are specifically enriched in NK cells. Notably, transcription factors such as KMT2A, MED1, BRD4, ERG, E2F6, MAF, HEY1, H2AFZ, STAT1, and CDK9 are highly expressed in NK cells (**Supplementary Figure S7D**). These factors are instrumental in the development and functionality of NK cells, with their expression alterations being intricately linked to tumorigenesis and tumor progression. Thus, NK cells appear to participate in the translocation of multiple signaling molecules in PDAC and subsequent immune response.

### 3.3 Establishing and affirming a survival model for PDAC centered on NK cell-related genes

**Figure 3:**
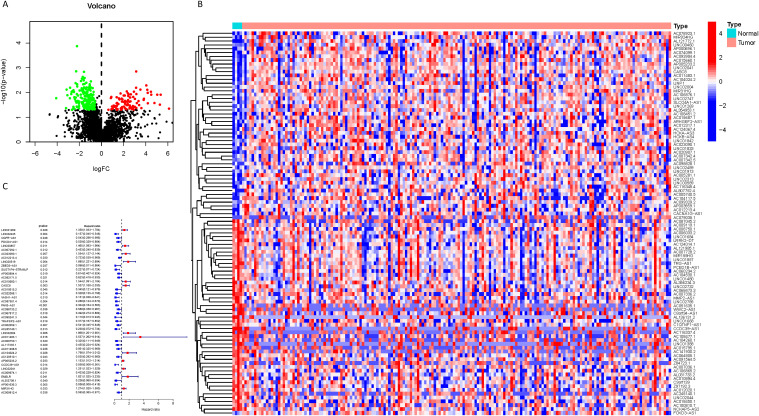
Identification of NK cell-related lncRNAs associated with prognosis. **(A)** A volcano plot displaying the 304 differentially expressed NK cell-related lncRNAs identified in PDAC (red: logFC > 0.585, FDR adjusted p < 0.05; green: logFC < 0.585, FDR adjusted p < 0.05); **(B)** A heatmap visually presenting the top 50 NK cell-related lncRNAs with the most significant expression differences; **(C)** A forest plot showing the results of univariate Cox regression analysis, identifying 42 lncRNAs associated with the prognosis of PDAC patients, where blue indicates a hazard ratio < 1 and red indicates a hazard ratio > 1.

**Figure 4:**
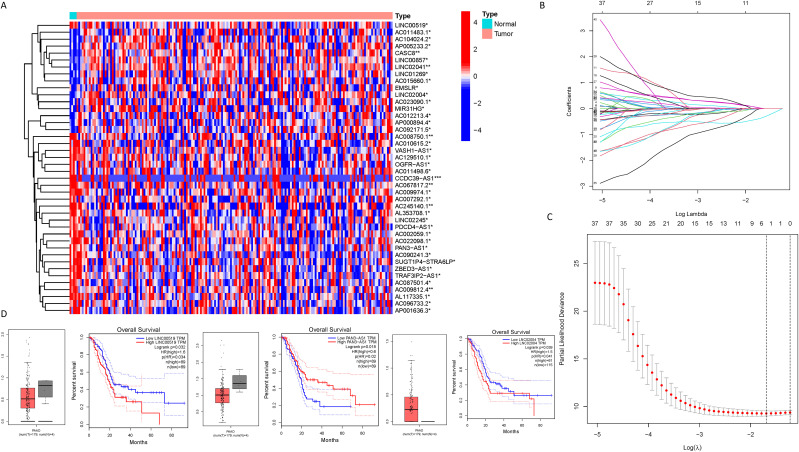
Identification of NK cell-related lncRNAs associated with prognosis. **(A)** A heatmap illustrating the expression differences of the lncRNAs identified in **(C)** between PDAC samples and normal samples (*** represents p < 0.001, ** represents p < 0.01, * represents p < 0.05); **(B, C)** Lasso regression analysis reveals the degree of overfitting in the model under different gene number settings and compares the severity of overfitting under these settings; **(D)** A box plot depicting the expression distribution of specific lncRNAs in the PDAC model from the TCGA database, and the results of survival analysis suggest a correlation between the expression levels of lncRNAs in the TCGA database and the clinical prognosis of PDAC patients.

**Figure 5:**
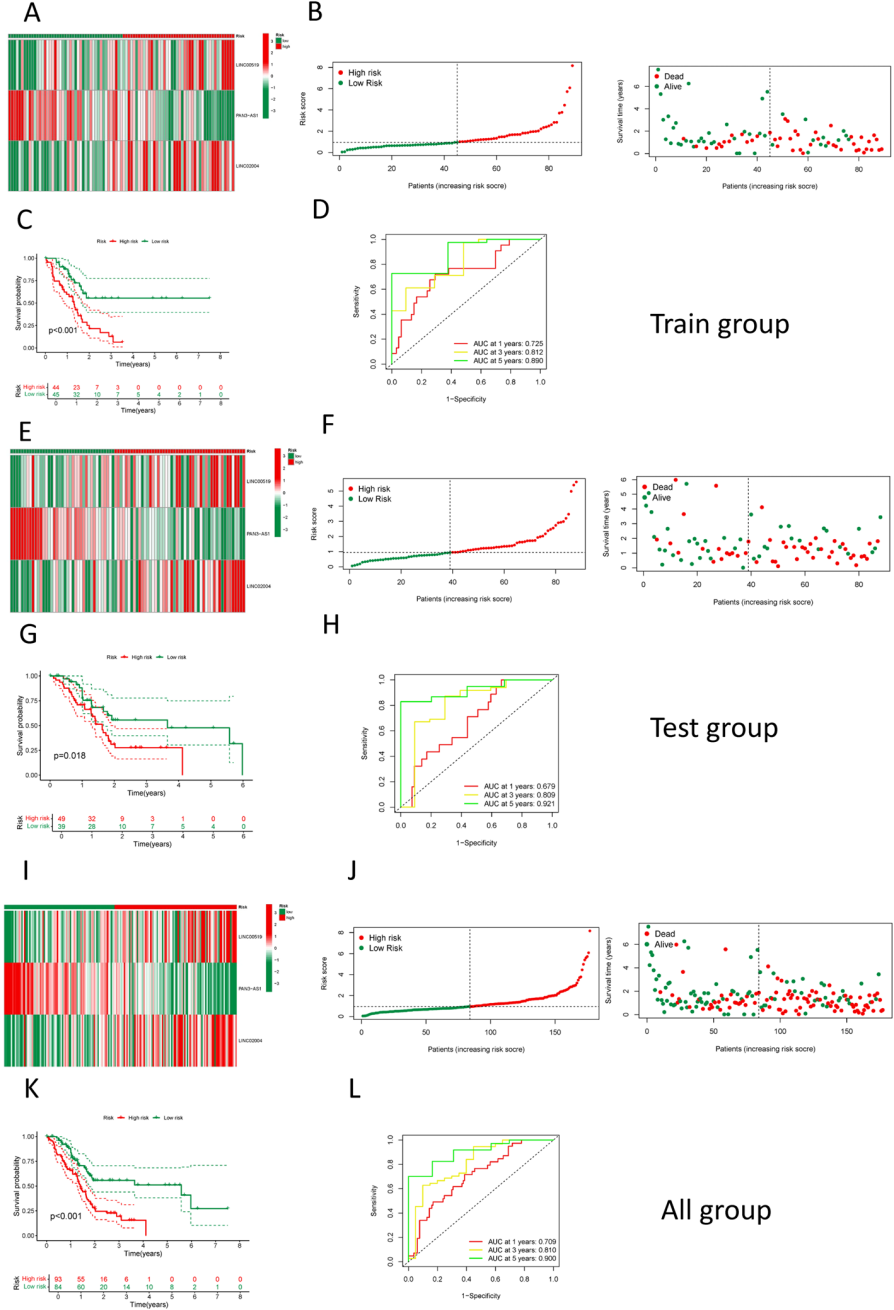
Construction and validation of the NK cell-related prognostic model. **(A)** A heatmap showing the expression of the 3 LncRNAs in high and low-risk groups in the training set; **(B)** The distribution and survival status of PDAC patients in the training set; **(C)** A comparison of survival curves between high and low-risk groups in the training set; **(D)** An assessment of the ROC curve in the training set; **(E)** A heatmap showing the expression of the 3 LncRNAs in high and low-risk groups in the test set; **(F)** The distribution and survival status of PDAC patients in the test set; **(G)** A comparison of survival curves between high and low-risk groups in the test set; **(H)** An assessment of the ROC curve in the test set; **(I)** A heatmap showing the expression of the 3 LncRNAs in high and low-risk groups among all patients; **(J)** The distribution and survival status of PDAC patients among all patients; **(K)** A comparison of survival curves between high and low-risk groups among all patients; **(L)** An assessment of the ROC curve among all patients.

As delineated in **Figure 1**, we initially extracted the gene expression patterns of the previously identified differential NK cell-related genes from the TCGA-PAAD dataset. Employing the Pearson correlation coefficient, we pinpointed NK cell-associated lncRNAs correlated with these genes (correlation coefficient > 0.4, p-value < 0.001). Through differential expression investigation, a total of 304 NK cell-related lncRNAs exhibiting differential expression in PDAC were identified (**Figure 3A**). A heatmap graphically represents the top 50 most significant DEGs among PDAC patients (**Figure 3B**). Subsequent univariate Cox regression research on the training dataset, preliminarily screening out 42 lncRNAs related to the prognosis of PDAC patients (**Figure 3C**), which served as the foundation for subsequent selection steps. The heatmap illustrated the expression variance of these lncRNAs between PDAC and normal samples (**Figure 4A**). To prevent model overfitting, LASSO regression analysis was applied (**Figures 4B, C**). Such a step facilitated the refinement of 42 candidate lncRNAs to identify key prognostic factors, thereby simplifying the model and enhancing its predictive precision. Utilizing the foundation laid by the initial steps, we proceeded with multivariate Cox regression analysis, a pivotal component in the construction of a clinical prognostic model. This analysis takes into account the interplay and influence among multiple variables, offering a more holistic predictive model. Through this process, we ultimately identified 3 lncRNAs that were significant. To validate the prognostic correlation of these three lncRNAs in PDAC patients, we utilized the GEPIA online analysis tool to visualize their expression levels. Box plots depicted the expression profiles of these lncRNAs among PDAC patients within the TCGA database, with survival analysis suggesting a potential link between their expression levels and PDAC prognosis (**Figure 4D**). A PDAC patient risk model was established using three specific NK-related lncRNAs: LINC00519, PAN3-AS1, and LINC02004. The risk score is derived using the following formula: risk score = 0.456120265000581 * expression of LINC00519 - 1.12039497572332 * expression of PAN3-AS1 + 0.479223014745653 * expression of LINC02004. The risk score was used to divide patients into two categories: low risk and high risk. **Figure 5A** displays the expression profiles that were compared between high-risk and low-risk groups in the training dataset. It can be observed that LINC00519 and LINC02004 are highly expressed in the high-risk group, while PAN3-AS1 is highly expressed in the low-risk group, which is consistent with the risk score coefficients (LINC00519 and LINC02004 being positive influencing factors, and PAN3-AS1 being a negative influencing factor). while The distribution and survival outcomes of PDAC patients in the training set are depicted in **Figure 5B**. Survival analysis from **Figure 5C** indicates that the high-risk population within the training group has a poorer prognosis. The ROC curve for the NK-related lncRNA model in the training set demonstrates good performance, with AUC scores of 0.725, 0.812, and 0.890 for 1, 3, and 5 years, respectively (**Figure 5D**). Validation using the test set and the whole dataset authenticated the level of lncRNAs connected to NK cells(**Figures 5E–L**), survival analysis, and differential analysis, demonstrating the robustness of the findings.

### 3.4 Clinical relevance analysis of the NK cell-associated lncRNA model

**Figure 6:**
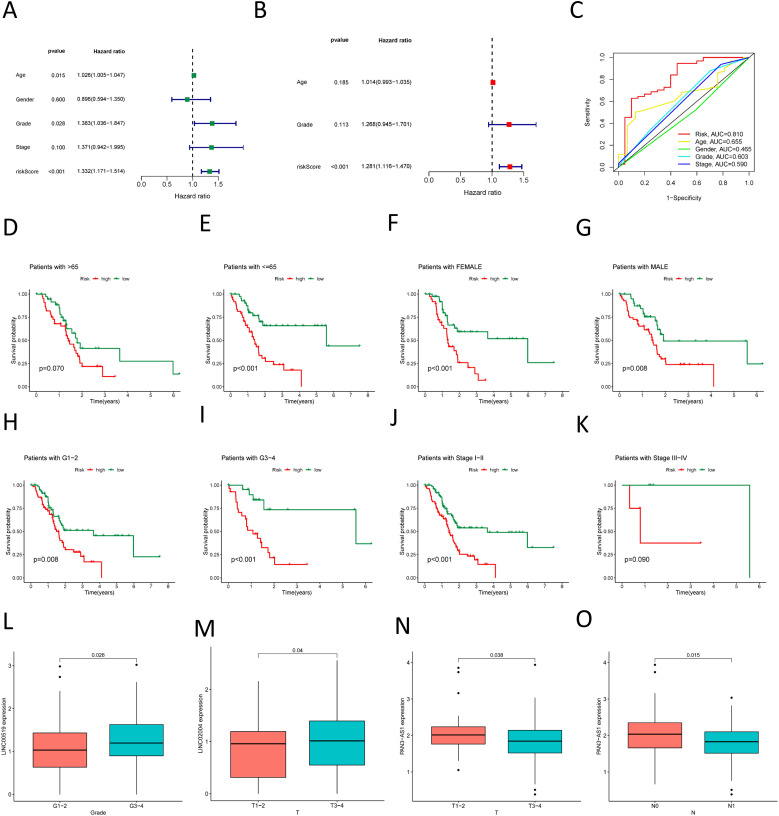
Association of the prognostic model with clinical factors. **(A)** Univariate COX regression demonstrates the factors affecting the prognosis of PDAC; **(B)** Multivariate COX regression shows the independent factors influencing the prognosis of PDAC; **(C)** ROC curve analysis assesses the accuracy of various clinical variables and risk scores in predicting the prognosis of PDAC; **(D–K)** Survival curves for various clinical subgroups based on risk scores. **(L)** High LINC00519 expression was associated with a worse Grade. **(M)** High LINC02004 expression was associated with a worse T-stage **(N, O)** Low PAN3-AS1 expression was associated with a worse T-stage and N-stage.

We employed the risk score as a prognostic variable for PDAC and conducted univariate COX regression analysis alongside other clinical parameters to evaluate the clinical utility of the NK cell-related lncRNA model. **Figure 6A** identifies age, Grade classification, and risk score as statistically significant prognostic factors for PDAC (hazard ratio > 1, p < 0.05). These predictors were then exposed to multivariate COX regression analysis, and the results indicated that only the risk score emerged as an independent prognostic indicator (**Figure 6B**). Moreover, ROC analysis demonstrated that among all these clinical parameters, the risk score’s evaluative efficacy significantly surpassed that of other factors (**Figure 6C**). We also conducted stratified analyses on the basis of different clinical features and found that the risk score had a good predictive effect across various subgroups, including gender, stage of grading, and age less than or equal to 65 years, as well as in early-stage patients (**Figures 6E–J**). In patients over the age of 65 and in late-stage (III-IV) disease, although a high-risk score seemed to be associated with a poorer prognosis, The connection observed was not statistically significant (P > 0.05), potentially on account of the sample size being too limited in these subgroups (**Figures 6D, K**). We also investigated the expression of these three lncRNAs in relation to clinical parameters and found that, among the most meaningful clinical grading and staging indicators related to prognosis, high LINC00519 expression was associated with a worse T-stage (**Figure 6L**), high LINC02004 expression was associated with a worse Stage (**Figure 6M**), and low PAN3-AS1 expression was associated with a worse T-stage and N-stage (**Figures 6N, O**). This is consistent with our research findings on the impact of lncRNAs on pancreatic cancer. Although other results did not yield positive outcomes, the trends were similar (**Supplementary Figure S8**). The main reason for the negative results may be related to the small sample size, and it is also possible that these three lncRNAs influence the progression of pancreatic cancer through other pathways.

### 3.5 Exploring pathway and enrichment differences among risk groups

**Figure 7:**
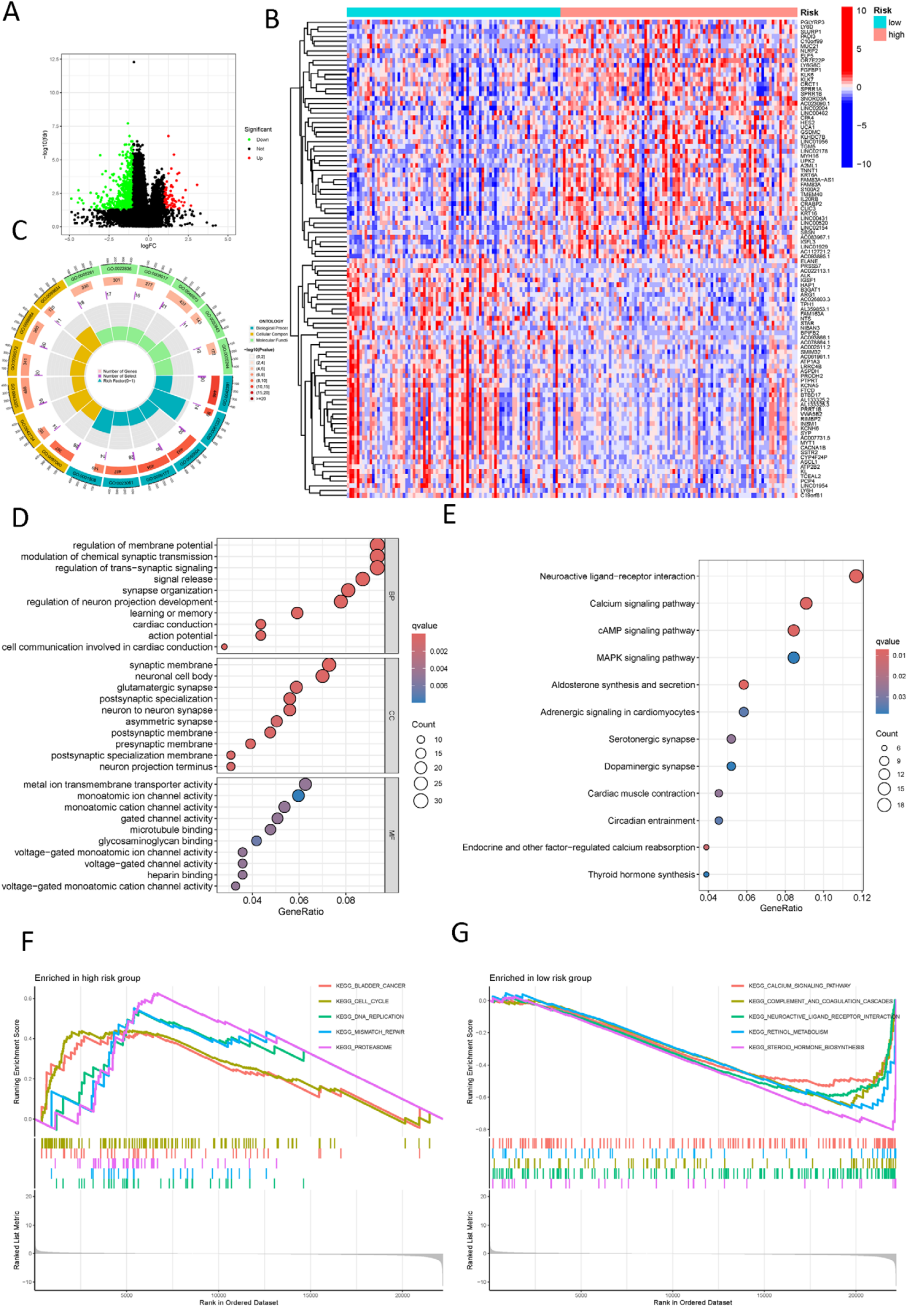
Functional enrichment analysis of different risk groups. **(A)** A volcano plot displays differentially expressed genes between different risk groups (red: logFC > 1, FDR adjusted p < 0.05; green: logFC < 1, FDR adjusted p < 0.05); **(B)** A heatmap shows the distribution of differential genes across different risk groups; **(C)** A Circos plot reveals the changes in differentially expressed genes in the graphene oxide pathways; **(D)** A bubble chart reveals the enrichment of graphene oxide signaling pathways with significantly differential gene expression; **(E)** A bubble chart shows the concentration of significantly differentially expressed genes in KEGG signaling pathways; **(F)** Displays the upregulated pathways in the high-risk group according to GSEA; **(G)** Displays the downregulated pathways in the low-risk group according to GSEA.

Seeking to explain the poorer prognosis in the high-risk group as opposed to the low-risk group, the differential analysis revealed 597 genes with significant differences in expression levels (|logFC| > 1, FDR adjusted p < 0.05) (**Figure 7A**). A heatmap was employed to visually represent the 50 most significant DEGs (**Figure 7B**). GO enrichment analysis revealed substantial changes in the high-risk group, particularly associated with the regulation of membrane potential and synaptic structure (**Figures 7C, D**). KEGG pathway enrichment revealed an overrepresentation of cell signaling-related processes in the high-risk cohort, containing neuroactive ligand-receptor interaction signaling pathways, calcium signaling pathways, cAMP signaling pathways, and MAPK signaling pathways (**Figure 7E**). Besides, the GSEA uncovered notable enrichment in molecular mechanisms associated with cell proliferation, DNA replication, and repair in the high-risk cohort, which may be linked to the tumor’s origin, development, and therapeutic responsiveness. However, the low-risk cohort exhibited a richer presence of pathways involved in metabolism and signaling, blood and immune responses, and hormone regulation, potentially relating to better physiological function and stronger disease defense capabilities (**Figures 7F, G**).

### 3.6 Somatic mutation analysis in different risk groups

**Figure 8:**
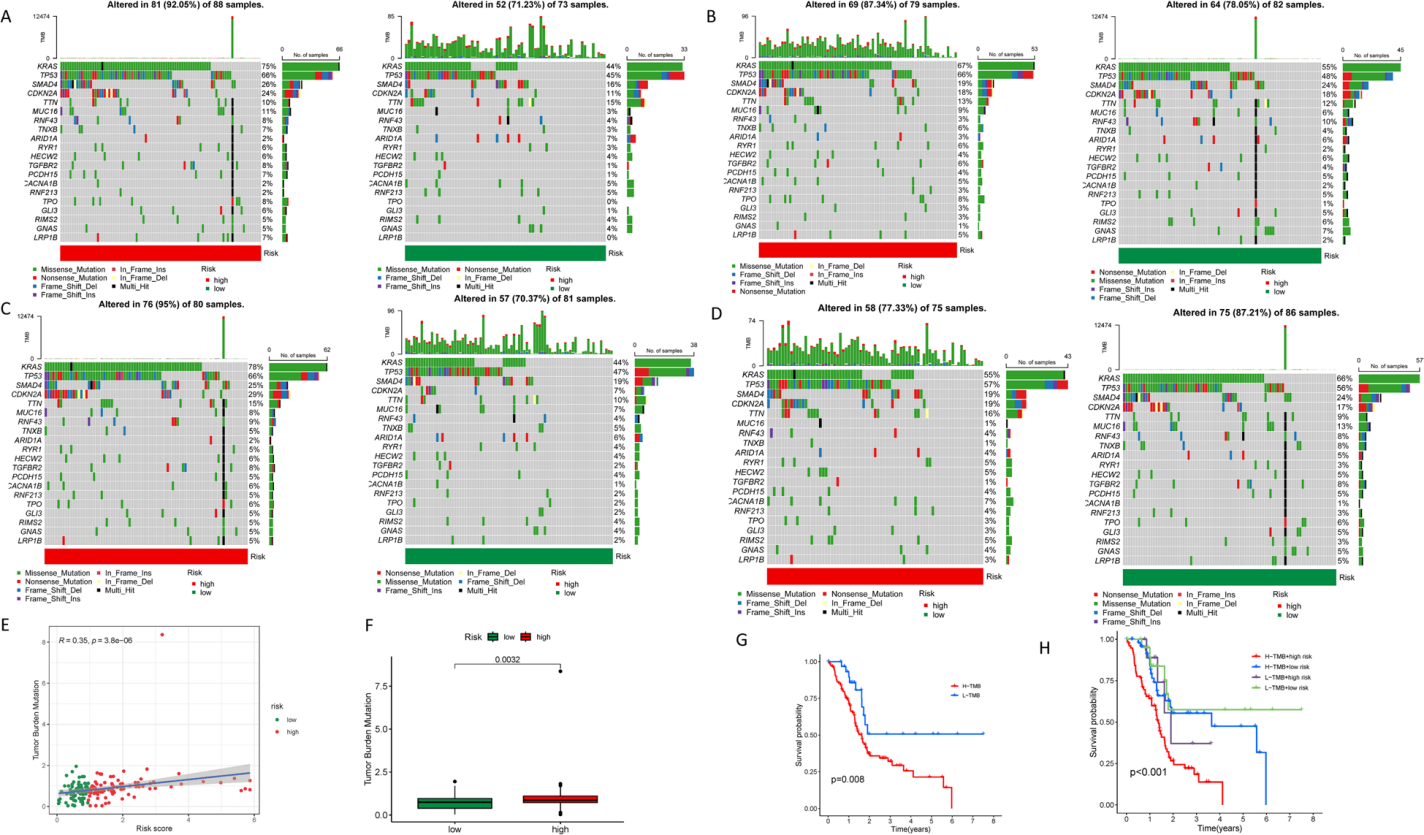
Tumor mutational burden in different risk groups. **(A)** Waterfall plots showing the genetic mutation status in high and low-risk groups; **(B)** Waterfall plots showing the genetic mutation status in high and low- LINC00519 expression groups; **(C)** Waterfall plots showing the genetic mutation status in high and low- LINC02004 expression groups; **(D)** Waterfall plots showing the genetic mutation status in high and low- PAN3-AS1 expression groups; **(E, F)** Correlation analysis between tumor mutational burden and risk scores; **(G)** Survival analysis revealing the prognosis of PDAC patients in different tumor mutational burden groups; **(H)** Survival analysis showing the impact of combining tumor mutational burden and risk scores on the prognosis of PDAC patients.

In the high-risk group, the three most frequently mutated genes are KRAS, TP53, and SMAD4. Notably, the mutation frequency of the KRAS gene in the high-risk cohort, at 75%, Is considerably elevated in contrast to the low-risk group, which is 44%. This disparity underscores the pivotal role of KRAS in the poorer prognosis associated with the high-risk cohort (**Figure 8A**). We further explored the relationship between LINC00519, PAN3-AS1, and LINC02004 and mutated genes, finding that in the group with high expression of LINC00519, there was a high mutation risk for KRAS and TP53. In the group with high expression of LINC02004, there was a high mutation risk for KRAS, TP53, and SMAD4. Conversely, in PAN3-AS1, which has a negative regulatory effect, the mutation rates of KRAS and SMAD4 were reduced. These results indicate a strong correlation between the expression levels of LINC00519, PAN3-AS1, and LINC02004 and the mutation frequencies of KRAS, TP53, and SMAD4 in PDAC (**Figures 8B–D**). Moreover, we assessed the tumor mutational burden (TMB) in PDAC patients, categorizing them into high and low TMB groups. An upward trend is observed between the risk score and TMB, with a high TMB correlating with an adverse prognosis (**Figures 8E, F**). High TMB is associated with poor survival. (**Figure 8G**) And it is particularly striking that patients with both high TMB and elevated risk scores exhibit the most severe prognosis, whereas those with low TMB and reduced risk scores have the most favorable outcomes. The combined effect of these two factors is more prominent than either TMB or risk score alone (**Figure 8H**).

### 3.7 Immune analysis in different risk groups

**Figure 9:**
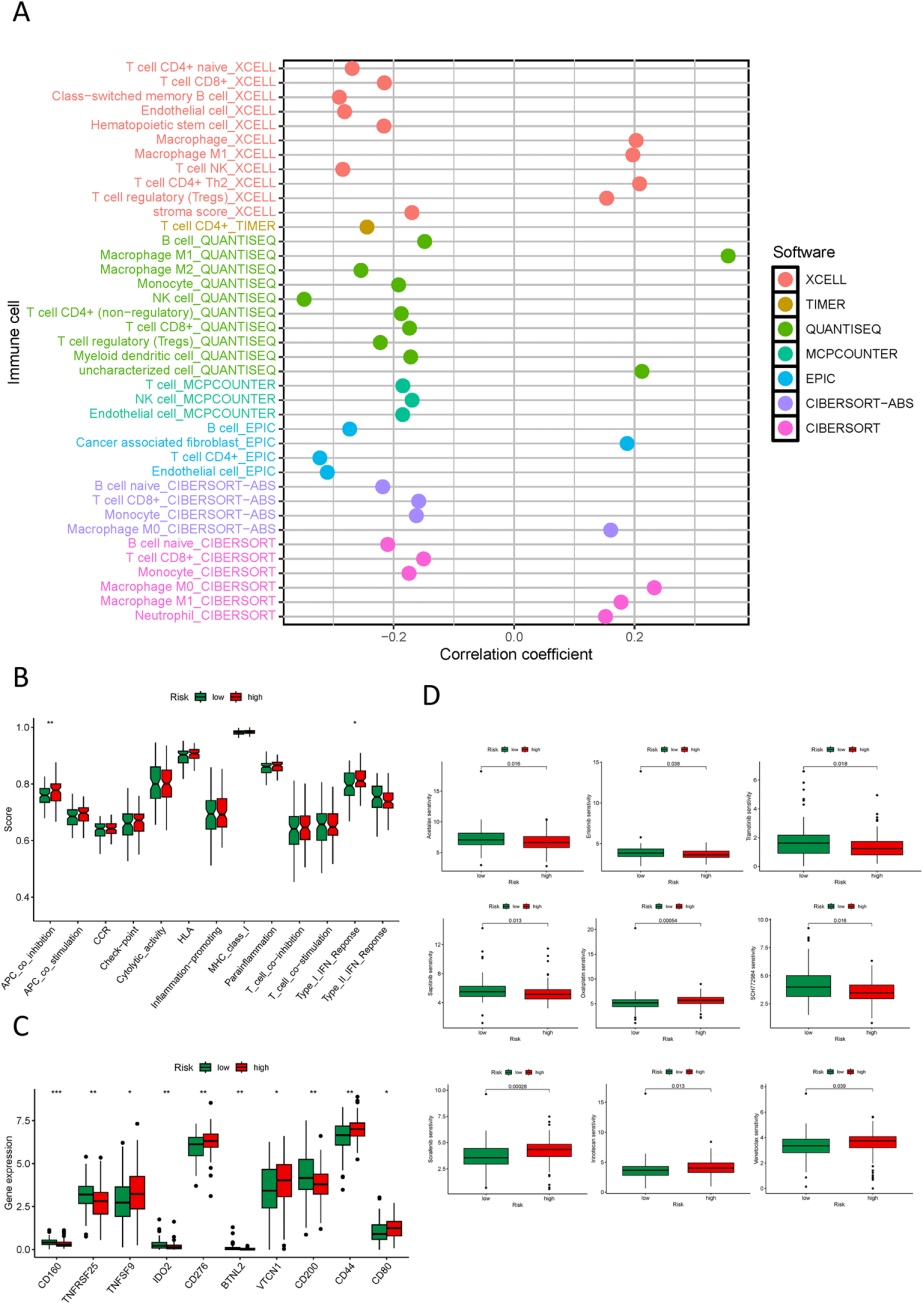
Immune, and drug sensitivity analysis in different risk groups. **(A)** Quantitative analysis of immune infiltration in PDAC using multiple algorithms; **(B)** Box plots displaying the immune function status in high and low-risk groups; **(C)** Box plots showing the immune checkpoint status in high and low-risk groups; **(D)** Box plots displaying the drug sensitivity status in high and low-risk groups. *p < 0.05, **p < 0.01, ***p < 0.001.

Our study delved into the relationship between the NK cell-connected lncRNA model score and immune infiltration in PDAC. Utilizing various software to analyze the immune cell infiltration status in PDAC samples, we discovered an inverse correlation between NK cell infiltration and the risk score. This suggests that within the tumor microenvironment, an increased level of NK cell infiltration corresponds to a lower risk score for patients, potentially indicating a more favorable prognosis (**Figure 9A**). The immune-related functional analysis between different risk groups demonstrated that the high-risk cohort displayed co-inhibition of antigen-presenting cells and activation of type I IFN response immune functions. This suggests that these patients may be in a state of immune suppression and could trigger a complex immune activation response (**Figure 9B**). The high level of immune checkpoint genes (ICGs) such as TNFSF9, CD276, VTCN1, CD44, and CD80 in the high-risk group, as well as TNFRSF25, CD200, CD160, IDO1, and BTNL2 in the low-risk cohort, suggest potential targets for corresponding immune checkpoint inhibitors (**Figure 9C**). In our drug sensitivity analysis, the high-risk group demonstrated a strong response to drugs like Acetalax, Erlotinib, Trametinib, and Sapitinib, indicating that this group may benefit more from these drugs. In contrast, low-risk cohort patients were preferable for drugs such as Irinotecan, Oxaliplatin, SCH772984, Sorafenib, and Venetoclax (**Figure 9D**; **Supplementary Figure S9**).

### 3.8 Characterization of PDAC molecular subtypes by NK cell-related lncRNA

**Figure 10:**
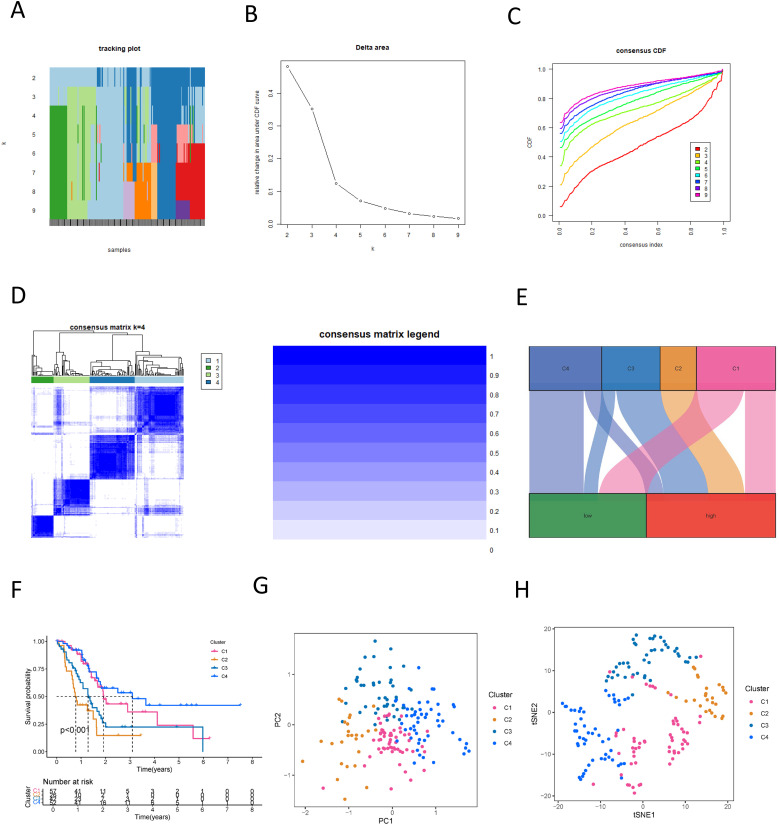
New subtyping of PDAC based on NK cell-related lncRNA. **(A)** Sample distribution with different numbers of subtypes; **(B)** CDF curves for different numbers of subtypes; **(C)** Consensus CDF for different numbers of subtypes; **(D)** Consensus matrix of the 4 subtypes; **(E)** A Sankey diagram showing the relationship between different PDAC subtypes and risk scores; **(F)** Survival curves for different PDAC subtypes; **(G)** PCA analysis reveals the distribution characteristics of samples in different PDAC subtypes; **(H)** t-SNE analysis presents the distribution of samples in various PDAC subtypes.

In our study, we utilized an NK cell-associated lncRNA model to classify PDAC tumor samples. Within the test set, κ = 4 demonstrated a more flattened and closer proximity to the maximum cumulative distribution function (CDF) (**Figures 10A–C**). Consequently, we set κ = 4 and categorized PDAC cancer specimens into four subtypes: Cluster 1-4 (C1, C2, C3, C4) (**Figure 10D**). A Sankey diagram illustrated the distribution of different risk score groups across these subtypes, with C1 showing an equal distribution, C2 being exclusively high-risk, C3 mainly high-risk, and C4 primarily low-risk (**Figure 10E**). Survival analysis revealed that C2, the cluster enriched with high-risk samples, exhibited the poorest prognosis (**Figure 10F**). This highlights the potential of the NK cell-related lncRNA model to stratify PDAC patients into clinically relevant molecular subtypes, which may inform personalized therapeutic strategies. Furthermore, we integrated principal component analysis (PCA) and t-distributed Stochastic Neighbor Embedding (tSNE) to validate the efficacy of our tumor classification approach, confirming the robustness of our clustering results (**Figures 10G, H**). These findings underscore the utility of the NK cell-related lncRNA model in delineating distinct subtypes of PDAC, affecting the future of precision medicine and the development of targeted therapeutics.

### 3.9 Immune analysis based on molecular subtypes defined by NK cell-related lncRNA

**Figure 11:**
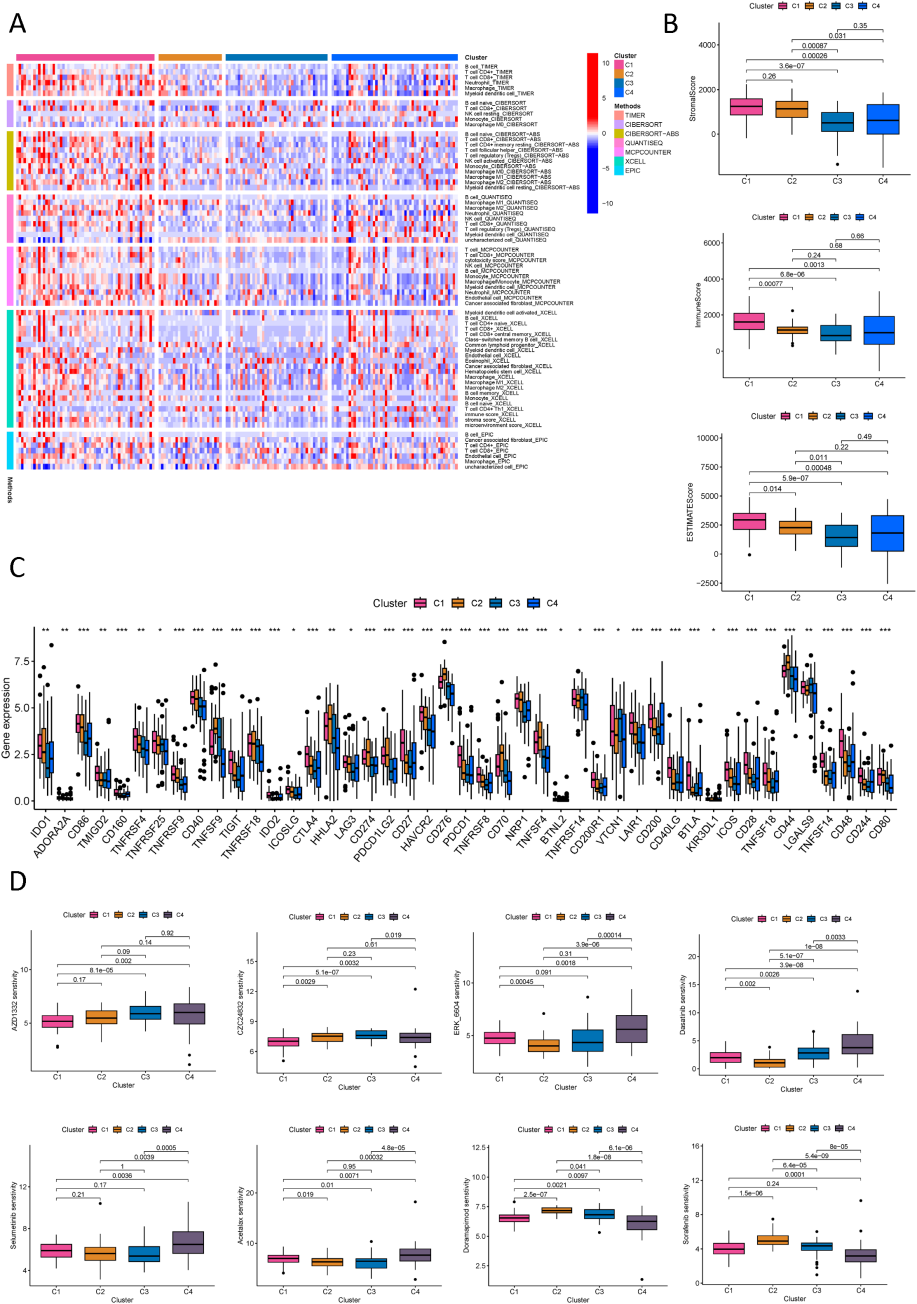
Immune and drug sensitivity analysis of PDAC new subtypes. **(A)** Quantitative analysis of immune infiltration in PDAC new subtypes using multiple algorithms; **(B)** ESTIMATE scores, stromal scores, and immune scores for different PDAC subtypes; **(C)** Analysis of immune checkpoints in different PDAC subtypes; **(D)** Analysis of drug sensitivity in different PDAC subtypes. *p < 0.05, **p < 0.01, ***p < 0.001.

To determine the utility of NK cell-related lncRNA molecular typing in immunotherapy for PDAC subtypes, an exhaustive analysis of the tumor immune microenvironment was conducted. Employing diverse computational algorithms, we identified that the C1 subtype of PDAC exhibited the most abundant immune cell infiltration, whereas the C3 subtype demonstrated the sparsest (**Figure 11A**). Further examination of the StromalScore, ImmuneScore, and ESTIMATEScore across subtypes revealed that the C3 subtype had the lowest scores, signifying more pronounced immune suppression in patients with the C3 subtype (**Figure 11B**). Concurrently, an immune checkpoint analysis was performed (**Figure 11C**), suggesting that PDAC subtypes with elevated immune checkpoint gene expression may benefit from targeted immune checkpoint inhibitors. Notably, the C2 subtype showed high expression of TNFSF9, HHLA2, CD274, PDCD1LG2, CD276, CD70, TNFSF4, CD44, and CD80; the C3 subtype featured high expression of TNFRSF14, VTCN1, and LGALS9; with the remaining genes predominantly highly expressed in the C1 subtype. The C4 subtype exhibited the lowest expression across most immune checkpoints, implying reduced immune suppression and a potentially better prognosis. Drug sensitivity analysis, stratified by the four molecular subtypes, indicated that C1 patients were most sensitive to AZD1332 and CZC24832; C2 patients to ERK_6604 and Dasatinib; C3 patients to Selumetinib and Acetalax; and C4 patients to Doramepimod and Sorafenib (**Figure 11D**; **Supplementary Figure S10**).

It can be seen that using molecular subtypes defined by NK cell-related LncRNA plays a role in evaluating the immune microenvironment and immunotherapy analysis, providing new ideas for further precision treatment of PDAC patients. An in-depth analysis of each subtype will help us achieve personalized treatment for PDAC patients and improve the efficacy of PDAC treatment.

### 3.10 Expression and functional validation of model lncRNAs in PDAC

**Figure 12:**
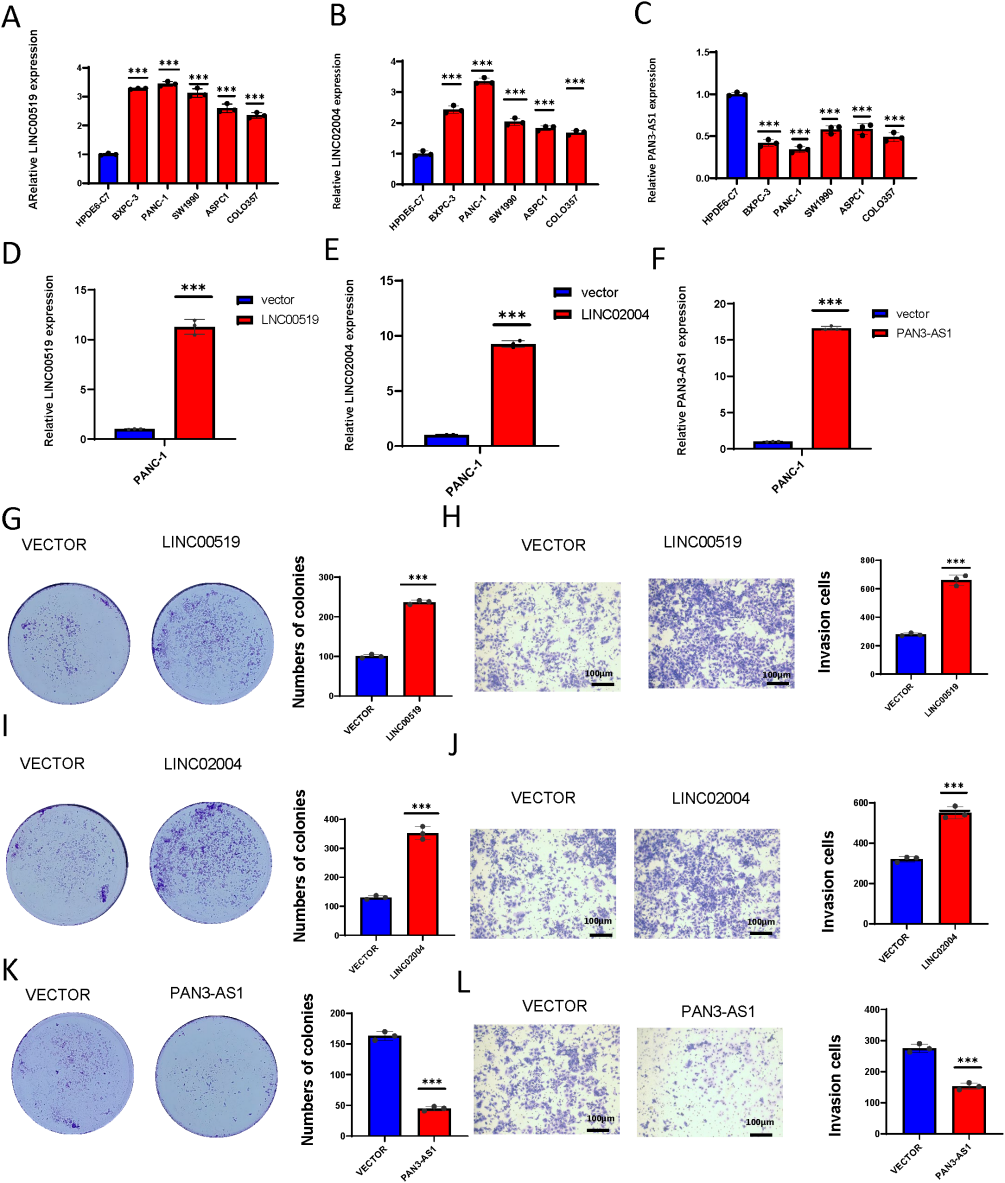
Expression and functional validation of model lncRNAs in PDAC. **(A)** The expression levels of LINC00519 vary across different pancreatic cell lines; **(B)** The expression levels of LINC02004 vary across different pancreatic cell lines; **(C)** The expression levels of PAN3-AS1 vary across different pancreatic cell lines; **(D)** Construct the LINC00519 overexpression plasmid; **(E)** Construct the LINC02004 overexpression plasmid; **(F)** Construct the PAN3-AS1 overexpression plasmid; **(G)** Colony formation assay to verify the proliferative capacity after overexpression of LINC00519; **(H)** Transwell assay to validate the invasive capacity after overexpression of LINC00519; **(I)** Colony formation assay to verify the proliferative capacity after overexpression of LINC02004; **(J)** Transwell assay to validate the invasive capacity after overexpression of LINC02004; **(K)** Colony formation assay to verify the proliferative capacity after overexpression of PAN3-AS1; **(L)** Transwell assay to validate the invasive capacity after overexpression of PAN3-AS1. ***p < 0.001.

To validate the roles of these three lncRNAs in PDAC, we first assessed their expression levels in the human normal cell line HPDE6-C7 and human pancreatic cancer cell lines BXPC-3, PANC-1, SW1990, ASPC1, and COLO357. We found that LINC00519 and LINC02004 were highly expressed in pancreatic cancer cell lines, while PAN3-AS1 was lowly expressed (**Figures 12A–C**). We further overexpressed these three lncRNAs in the PANC-1 cell line (**Figures 12D–F**) and discovered that overexpression of LINC00519 and LINC02004 increased the proliferative and invasive capabilities of pancreatic cancer cells, whereas overexpression of PAN3-AS1 diminished these capabilities (**Figures 12G–L**). Through experimental validation, we confirmed that LINC00519 and LINC02004 promote the progression of PDAC, while PAN3-AS1 may play a role in inhibiting its progression.

## 4 Discussion

While our comprehension of T cells in tumor immunity has significantly progressed, the function of NK cells in PDAC is an area that warrants further exploration. NK cells, in distinction to B and T cells, have receptors that can activate or inhibit cellular responses, with the balance between these signals determining their reactivity to target cells. In the TME, NK cells are attracted from the circulation to the tumor site in response to inflammatory chemokines. Once in the TME, NK cells rely on a “missing self” mechanism to detect and combat cancerous cells, and NK cells are capable of identifying and reacting to cells that exhibit “missing self” characteristics, which may ultimately lead to the elimination of the target cells that are detected and engaged by NK cells in response to these “missing self” cells, which may ultimately result in the destruction of the cells of interest (20). The “missing self” mechanism is vital for overcoming tumor cells that evade T-cell surveillance. Our investigation into NK cells within the TME revealed their enrichment in several critical signaling pathways, such as NK cell cytotoxicity, primary immunodeficiency, and antigen presentation, as well as T cell receptor signaling. We also noted significant enrichment in molecular functions involving cytoplasmic translation, ribosomal structures, and antigen binding, among others. These less-studied areas provide critical insights into the part played by NK cells in the realm of cancer immunology.

Studies continue to highlight the significant part that lncRNAs contribute to the regulation of immune cells (21). We identified key NK cell-related lncRNAs through correlation analysis and established a prognostic model based on three: LINC00519, LINC02004, and PAN3-AS1. LINC00519 and LINC02004 are high-risk indicators, while PAN3-AS1 suggests a low-risk profile. LINC02004’s overexpression in PDAC aligns with poor prognosis, whereas PAN3-AS1’s underexpression correlates with better outcomes. Interestingly, LINC00519’s underexpression in PDAC is still linked to poor prognosis, suggesting unknown regulatory complexities. These lncRNAs are understudied in pancreatic cancer, offering the potential for new therapeutic targets. Therefore, we experimentally verified the expression levels of these three lncRNAs and reached conclusions similar to those of our model. Furthermore, our experimental validation revealed that the expression of these lncRNAs is associated with the proliferation and invasion of PDAC. Stratifying patients by risk score, we found differential gene expression. Our findings revealed that these LncRNAs are predominantly engaged in signal transduction and the advancement of PDAC. For example, the neuroactive ligand-receptor interaction signaling pathway, calcium signaling pathway, cAMP signaling pathway, and MAPK signaling pathway in KEGG and the enrichment of molecular mechanisms related to cell proliferation, DNA replication, and repair in GSEA analysis. These findings elucidate the mechanisms of NK cell-associated LncRNAs in PDAC. Furthermore, our research has brought to light several under-explored and uncharted pathways in NK cells, including those involved in membrane potential regulation, synaptic structure, blood and immune responses, and hormone regulation. The identification of these pathways will steer our future investigations into the realm of NK cell-associated LncRNAs in tumor immunity.

Our study revealed an inverse correlation between NK cell infiltration and risk score, hinting at a possible association with improved prognosis. NK cells in peripheral blood readily engage blood tumor cells, but their infiltration into solid tumors is impeded by the dense tumor structure and the suppressive tumor microenvironment, which can induce NK cell exhaustion. This underscores the importance of investigating methods to restore and enhance NK cells’ tumor-killing capabilities. To this end, researchers are exploring diverse strategies to augment NK cell efficacy, crucial for advancing immunotherapies against solid tumors (22). Among emerging strategies, CAR-modified cell therapies are particularly promising. Despite this, CAR-T cell therapies encounter challenges such as treatment-related toxicity, production complexities, a lack of specific tumor antigens, and inadequate tumor infiltration. In contrast, CAR-NK cells present advantages like reduced toxicity, easier preparation, and a multiplicity of tumor-targeting pathways, positioning them as a superior alternative (23). While NK cell therapy shows potential in oncological treatments, it is not without challenges. Key issues include enhancing the survival and persistence of NK cells within tumors, accurately identifying tumor cells, streamlining cell preparation for scalability and standardization, and addressing the variability in clinical trial results. Addressing these hurdles is crucial for the advancement of NK cell therapy in cancer treatment (19). Further research aims to overcome these limitations to optimize the effects of NK cell therapy.

Our analysis of ICG expression between different risk groups identified significant dissimilarities. High-risk groups exhibited increased expression of ICGs such as TNFSF9, CD276, VTCN1, CD44, and CD80, whereas TNFRSF25, CD200, CD160, IDO1, and BTNL2 were more highly expressed in low-risk groups. These genes are integral to tumor immunity. Notably, TNFSF9 (CD137L) is a key activating immune checkpoint molecule, and its agonist, in combination with PD-L1, can potently activate and expand tumor-specific cytotoxic T cells, thereby enhancing their inhibitory and lethal effects on tumors (24). CD276 (B7-H3), an immune checkpoint molecule, is often overexpressed in various tumors and correlates with poor prognosis (25). The expression of other immune checkpoint molecules also hints at their potential role in the development of immune checkpoint inhibitors for PDAC. Validation of these roles will necessitate extensive, collaborative, multicenter trials.

Our clinical prediction model for PDAC is anchored in the differential expression of NK cell genes and constructed utilizing LncRNAs associated with NK cells as a prognostic framework, predicts a poorer prognosis for members of the high-risk subset. We confirmed the model’s accuracy through risk plotting, heatmap generation, ROC testing, and Kaplan-Meier survival analysis. The model emerged as a significant predictor in COX regression analyses, outperforming traditional clinical variables in ROC curve assessments. Validation with both test and complete datasets ensured the robustness and reproducibility of our findings.

In the realm of personalized medicine, precision in tumor subtype differentiation is crucial. Our risk score-based categorization of PDAC patients into four subtypes reveals distinct prognostic outcomes: C2, all high-risk, has the poorest prognosis; C4, largely low-risk, the best. The C1 subtype of PDAC exhibited the most robust immune cell infiltration, indicating a potential state of immune exhaustion. Conversely, the C3 subtype demonstrated the weakest immune cell infiltration, which may imply a diminished capacity of the immune system to effectively surveil and combat the tumor. The differential drug sensitivities across subtypes are vital for precise drug selection. This molecular subtype classification is instrumental in advancing PDAC’s precision treatment.

## 5 Conclusion

We introduce a groundbreaking clinical prediction model for PDAC that elucidates the characteristic role of NK cells, enabling a new molecular classification framework for patients. At the same time, we conducted validation for lncRNAs associated with NK cells. This advancement is set to significantly bolster the field of personalized and precision medicine.

## Data Availability

The original contributions presented in the study are included in the article/**Supplementary Material**. Further inquiries can be directed to the corresponding authors.

## Supplementary material

Supplementary Figure 1
**(A, B)** Descriptions of the interaction probabilities between NK cells, acting as donors and receptors, and specific gene pairs in other cells through Cell Chat.

Supplementary Figure 2 A heatmap visually displaying the upregulated immune gene sets across different cell subsets.

Supplementary Figure 3 A heatmap visually depicting the enriched Kyoto Encyclopedia of Genes and Genomes (KEGG) pathways upregulated by various cell subsets.

Supplementary Figure 4 A heatmap visually depicting the enriched KEGG pathways downregulated by various cell subsets.

Supplementary Figure 5
**(A)** A heatmap visually showing the enriched Gene Ontology biological process (GOBP) pathways upregulated in different cell subsets; **(B)** A heatmap visually showing the enriched GOBP pathways downregulated in different cell subsets.

Supplementary Figure 6
**(A)** A heatmap visually showing the enriched Gene Ontology cellular component (GOCC) pathways upregulated in different cell subsets; **(B)** A heatmap visually showing the enriched GOCC pathways downregulated in different cell subsets.

Supplementary Figure 7
**(A)** A heatmap visually showing the enriched Gene Ontology molecular function (GOMF) pathways upregulated in different cell subsets; **(B)** A heatmap visually showing the enriched GOMF pathways downregulated in different cell subsets. **(C)** A heatmap showing the differential expression of core transcription factors in various cells of PDAC; **(D)** A dot plot revealing transcription factors that are significantly expressed in NK cells of PDAC.

Supplementary Figure 8 Box plots demonstrating the relationship between model lncRNA molecules and clinical parameters.

Supplementary Figure 9 Box plot showing drug sensitivity in different risk groups.

Supplementary Figure 10 Box plots demonstrating drug sensitivity for different molecular typologies.
