# Supplementary figures and images for: Effects of NK cell-related lncRNA on the immune microenvironment and molecular subtyping for pancreatic ductal adenocarcinoma
Source: Front Immunol. 2025 Jan 13;15:1514259. doi: 10.3389/fimmu.2024.1514259 (PMC11770056; doi:10.3389/fimmu.2024.1514259)

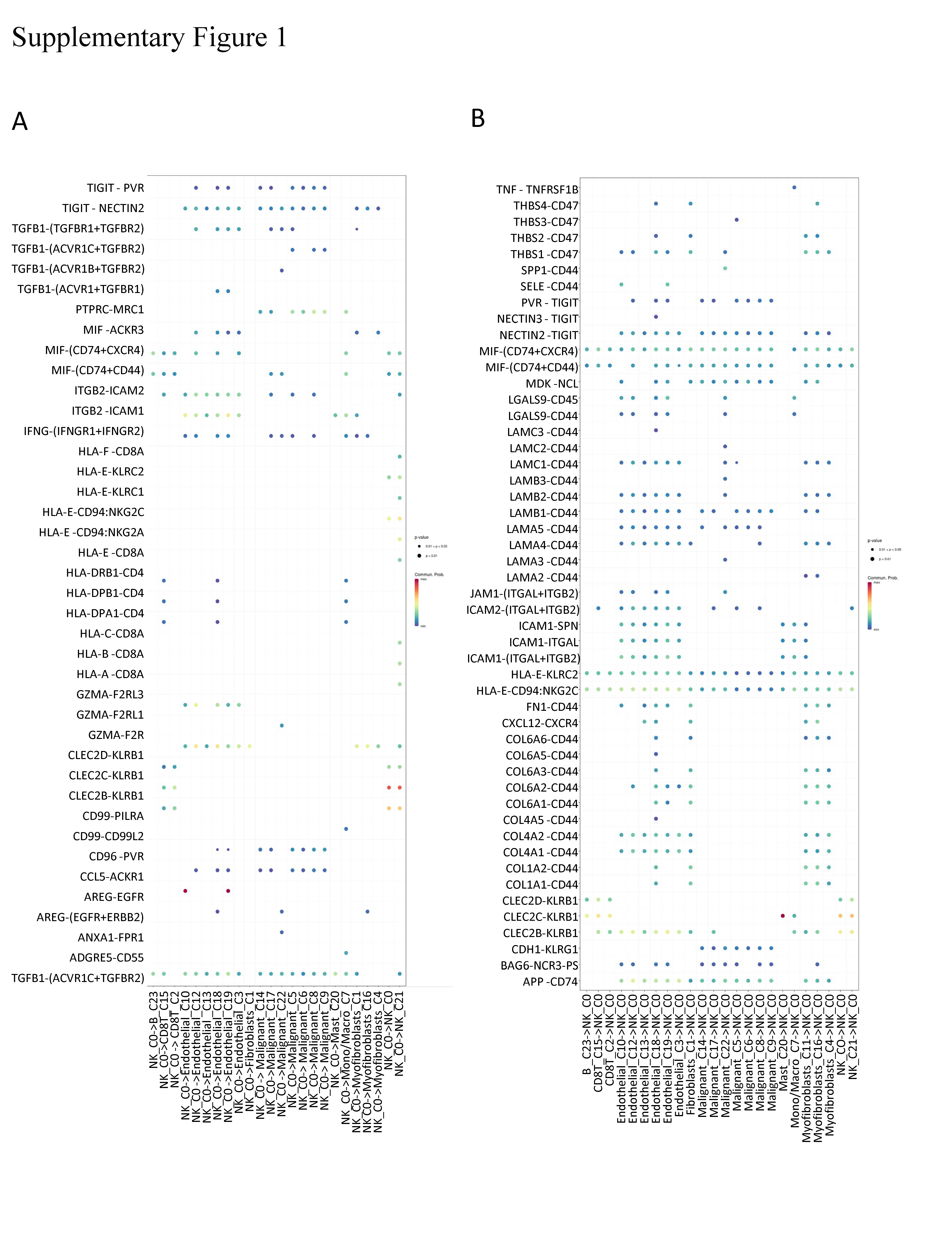

Supplement: Supplementary Figure 1 — (A, B) Descriptions of the interaction probabilities between NK cells, acting as donors and receptors, and specific gene pairs in other cells through Cell Chat. [file Image1.tif]

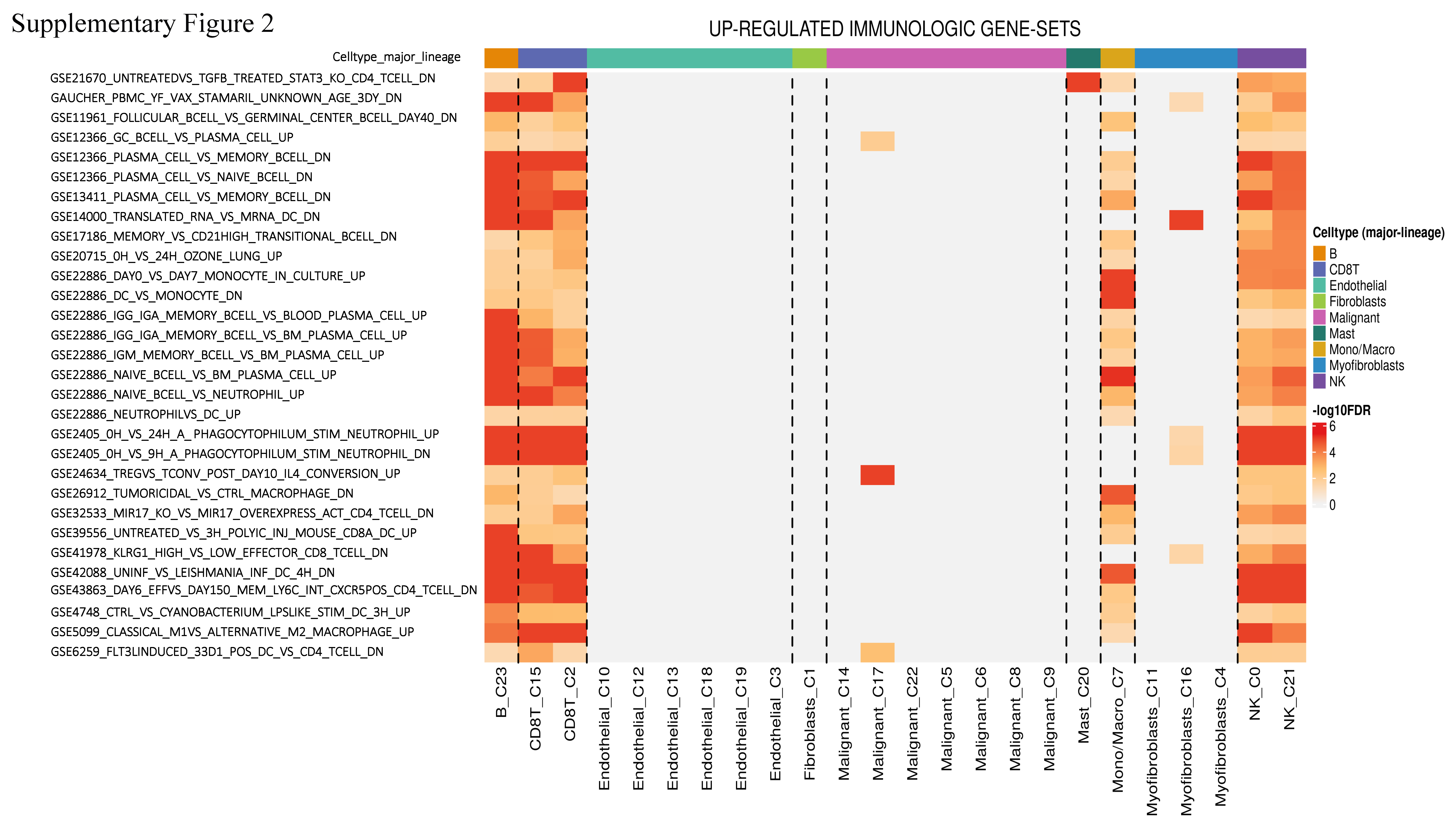

Supplement: Supplementary Figure 2 — A heatmap visually displaying the upregulated immune gene sets across different cell subsets. [file Image2.tif]

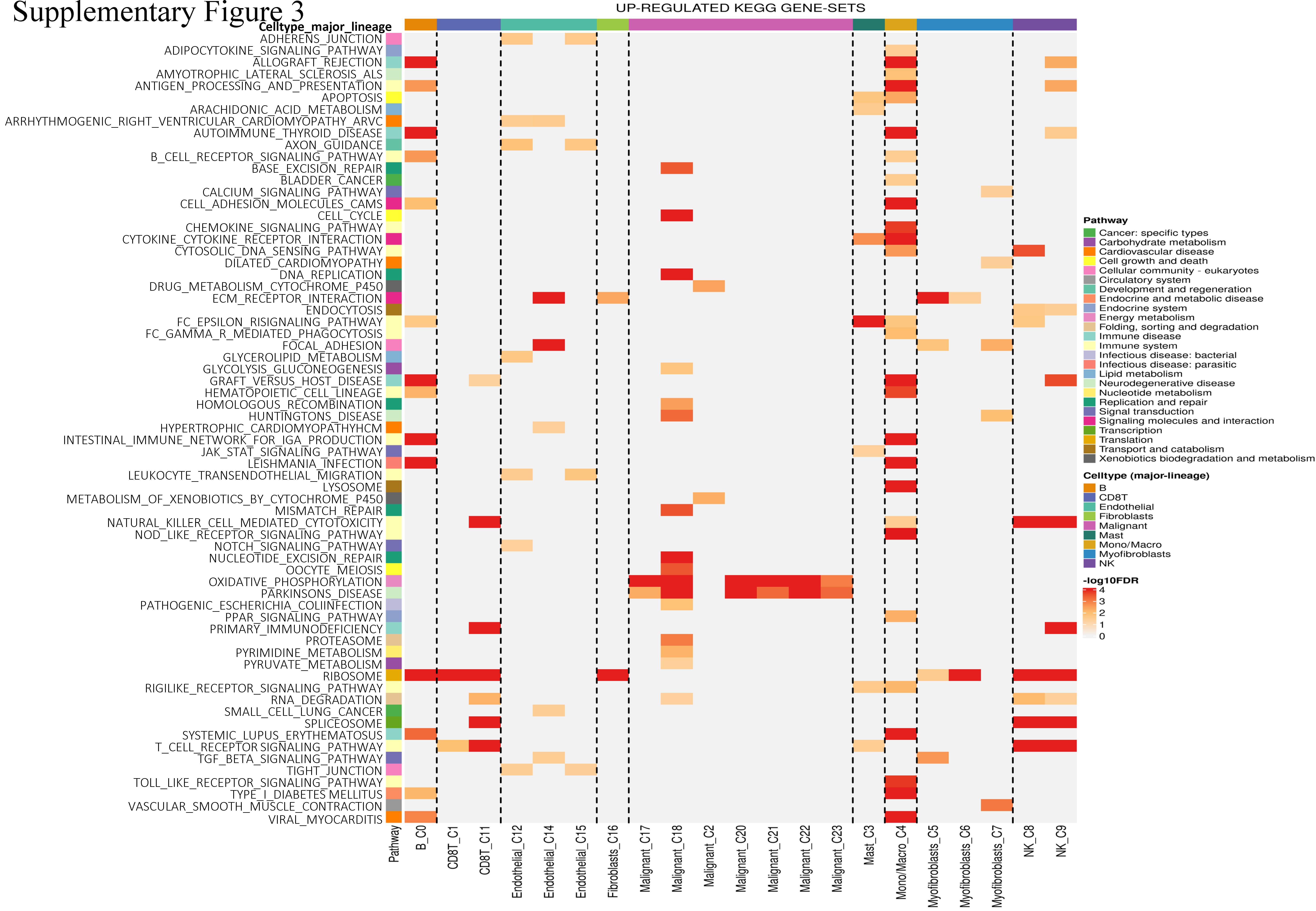

Supplement: Supplementary Figure 3 — A heatmap visually depicting the enriched Kyoto Encyclopedia of Genes and Genomes (KEGG) pathways upregulated by various cell subsets. [file Image3.tif]

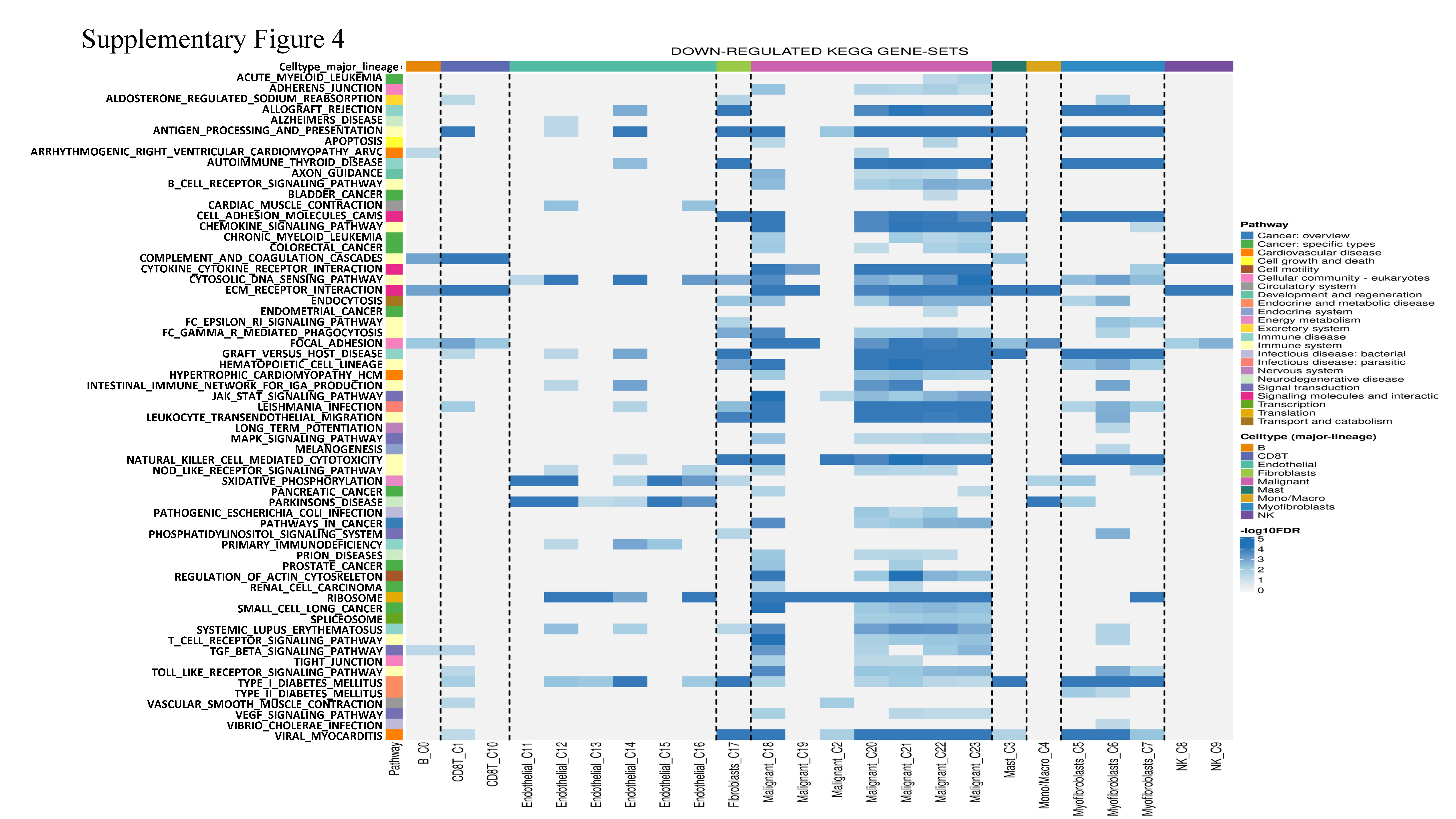

Supplement: Supplementary Figure 4 — A heatmap visually depicting the enriched KEGG pathways downregulated by various cell subsets. [file Image4.tif]

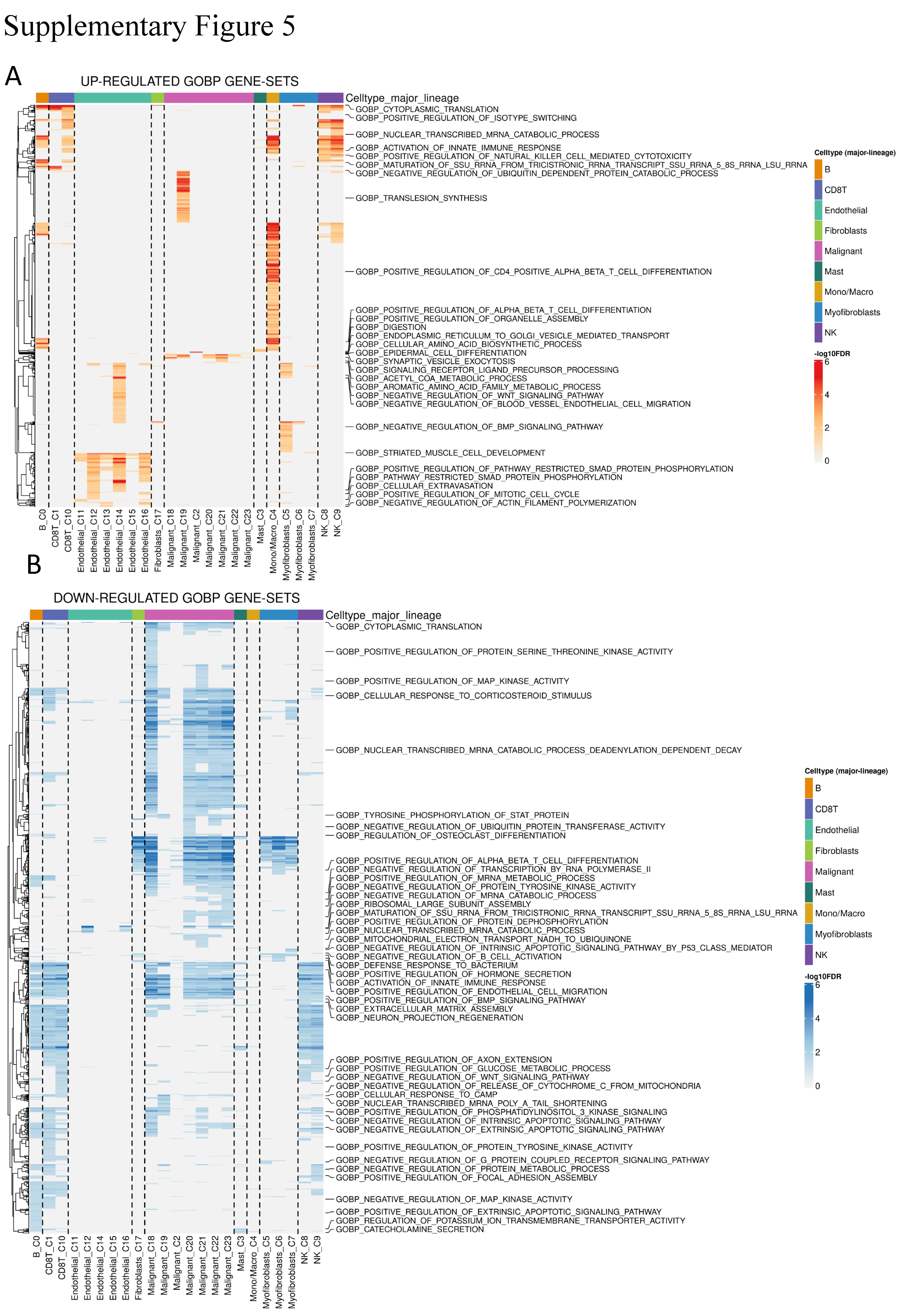

Supplement: Supplementary Figure 5 — (A) A heatmap visually showing the enriched Gene Ontology biological process (GOBP) pathways upregulated in different cell subsets; (B) A heatmap visually showing the enriched GOBP pathways downregulated in different cell subsets. [file Image5.tif]

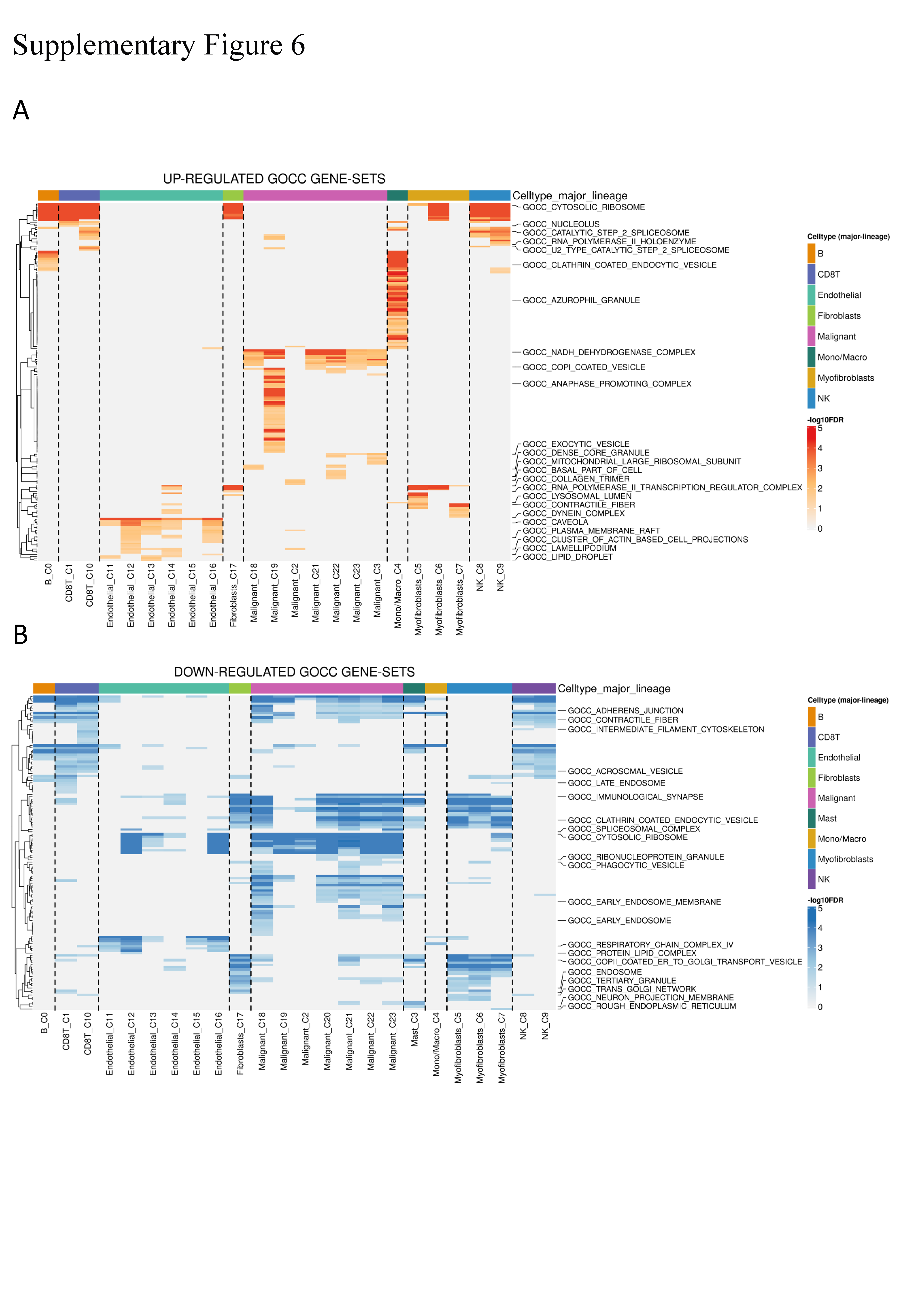

Supplement: Supplementary Figure 6 — (A) A heatmap visually showing the enriched Gene Ontology cellular component (GOCC) pathways upregulated in different cell subsets; (B) A heatmap visually showing the enriched GOCC pathways downregulated in different cell subsets. [file Image6.tif]

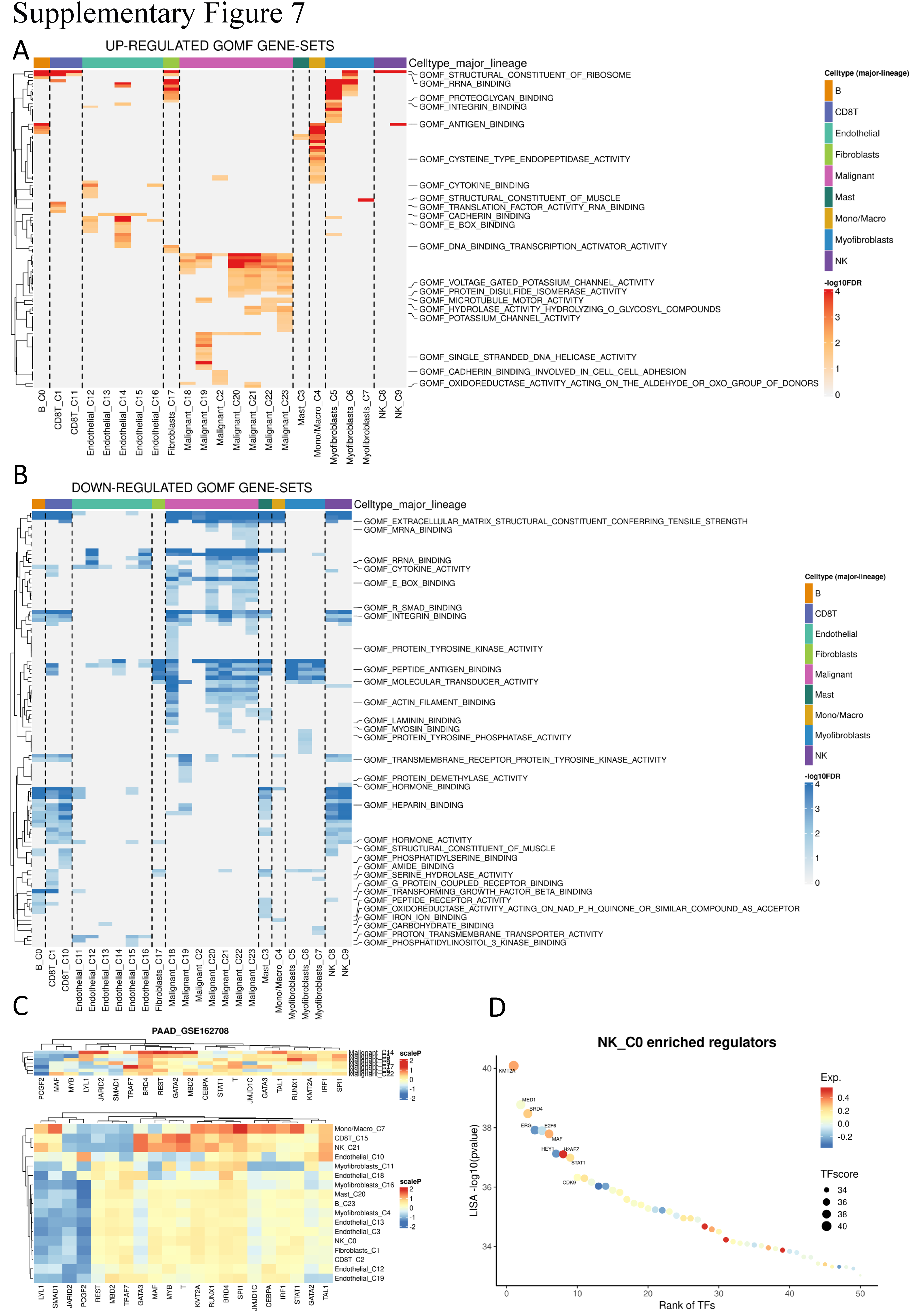

Supplement: Supplementary Figure 7 — (A) A heatmap visually showing the enriched Gene Ontology molecular function (GOMF) pathways upregulated in different cell subsets; (B) A heatmap visually showing the enriched GOMF pathways downregulated in different cell subsets. (C) A heatmap showing the differential expression of core transcription factors in various cells of PDAC; (D) A dot plot revealing transcription factors that are significantly expressed in NK cells of PDAC. [file Image7.tif]

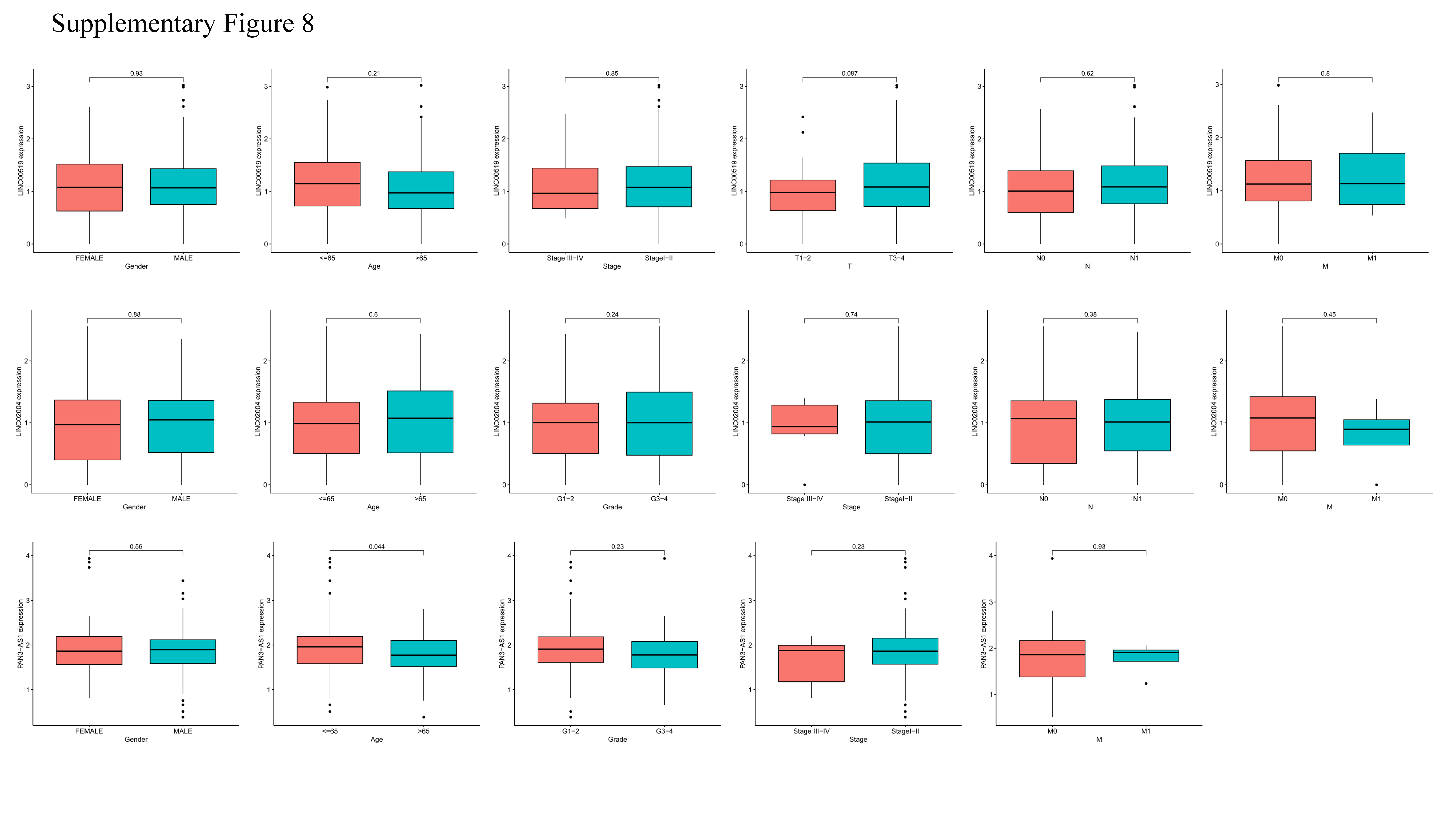

Supplement: Supplementary Figure 8 — Box plots demonstrating the relationship between model lncRNA molecules and clinical parameters. [file Image8.tif]

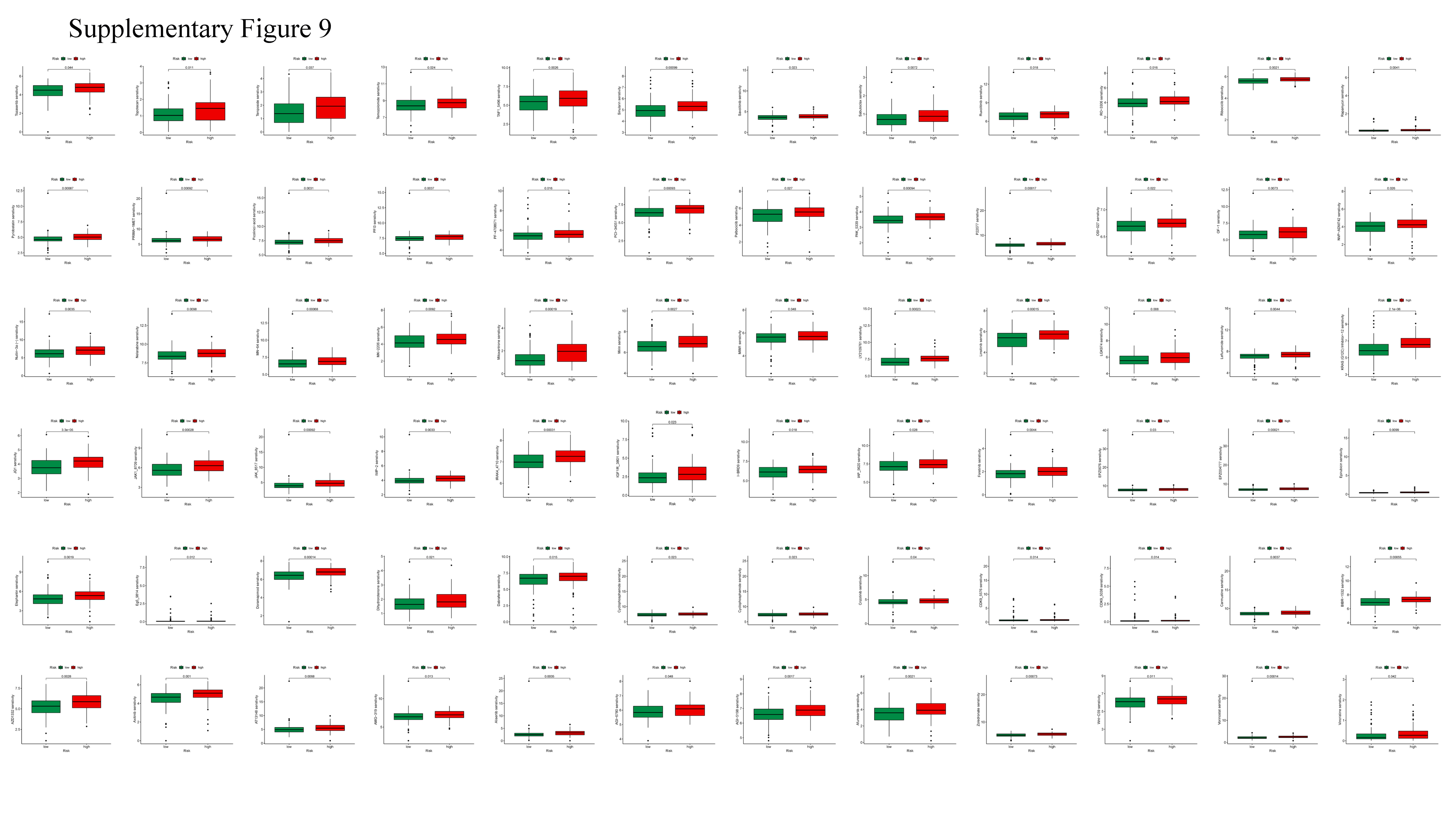

Supplement: Supplementary Figure 9 — Box plot showing drug sensitivity in different risk groups. [file Image9.tif]

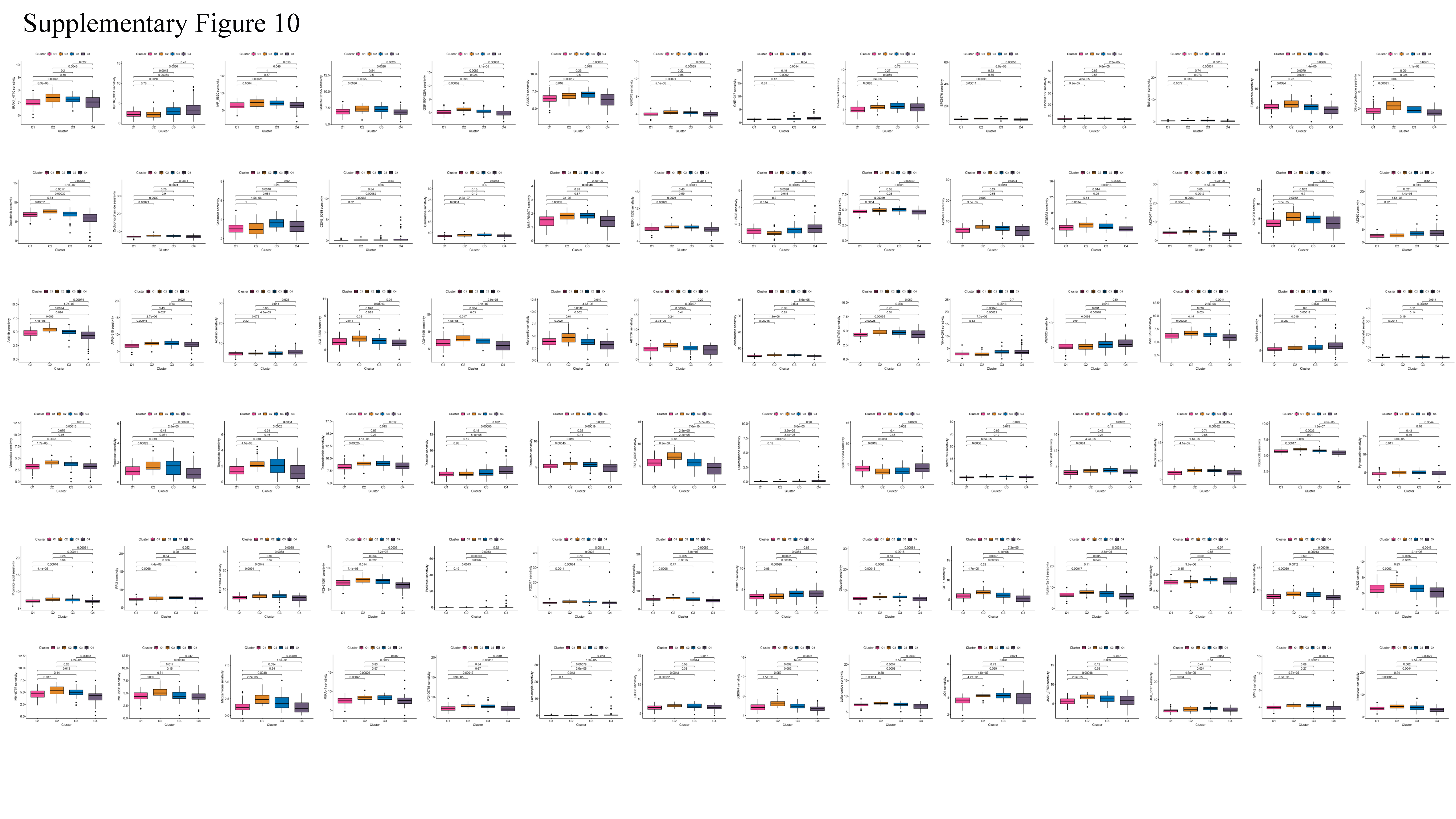

Supplement: Supplementary Figure 10 — Box plots demonstrating drug sensitivity for different molecular typologies. [file Image10.tif]
